# The impact of founder personalities on startup success

**DOI:** 10.1038/s41598-023-41980-y

**Published:** 2023-10-17

**Authors:** Paul X. McCarthy, Xian Gong, Fabian Braesemann, Fabian Stephany, Marian-Andrei Rizoiu, Margaret L. Kern

**Affiliations:** 1 The Data Science Institute, University of Technology Sydney, Sydney, NSW Australia; 2 School of Computer Science and Engineering, UNSW Sydney, Sydney, NSW Australia; 3 Faculty of Engineering and Information Technology, University of Technology Sydney, Sydney, Australia; 4 Oxford Internet Institute, University of Oxford, Oxford, UK; 5 DWG Datenwissenschaftliche Gesellschaft Berlin, Berlin, Germany; 6 Melbourne Graduate School of Education, The University of Melbourne, Parkville, VIC Australia

**Keywords:** Human behaviour, Information technology

## Abstract

Startup companies solve many of today’s most challenging problems, such as the decarbonisation of the economy or the development of novel life-saving vaccines. Startups are a vital source of innovation, yet the most innovative are also the least likely to survive. The probability of success of startups has been shown to relate to several firm-level factors such as industry, location and the economy of the day. Still, attention has increasingly considered internal factors relating to the firm’s founding team, including their previous experiences and failures, their centrality in a global network of other founders and investors, as well as the team’s size. The effects of founders’ personalities on the success of new ventures are, however, mainly unknown. Here, we show that founder personality traits are a significant feature of a firm’s ultimate success. We draw upon detailed data about the success of a large-scale global sample of startups (n = 21,187). We find that the Big Five personality traits of startup founders across 30 dimensions significantly differ from that of the population at large. Key personality facets that distinguish successful entrepreneurs include a preference for variety, novelty and starting new things (openness to adventure), like being the centre of attention (lower levels of modesty) and being exuberant (higher activity levels). We do not find one ’Founder-type’ personality; instead, six different personality types appear. Our results also demonstrate the benefits of larger, personality-diverse teams in startups, which show an increased likelihood of success. The findings emphasise the role of the diversity of personality types as a novel dimension of team diversity that influences performance and success.

## Introduction

The success of startups is vital to economic growth and renewal, with a small number of young, high-growth firms creating a disproportionately large share of all new jobs^1, 2^. Startups create jobs and drive economic growth, and they are also an essential vehicle for solving some of society’s most pressing challenges.

As a poignant example, six centuries ago, the German city of Mainz was abuzz as the birthplace of the world’s first moveable-type press created by Johannes Gutenberg. However, in the early part of this century, it faced several economic challenges, including rising unemployment and a significant and growing municipal debt. Then in 2008, two Turkish immigrants formed the company BioNTech in Mainz with another university research colleague. Together they pioneered new mRNA-based technologies. In 2020, BioNTech partnered with US pharmaceutical giant Pfizer to create one of only a handful of vaccines worldwide for Covid-19, saving an estimated six million lives^3^. The economic benefit to Europe and, in particular, the German city where the vaccine was developed has been significant, with windfall tax receipts to the government clearing Mainz’s €1.3bn debt and enabling tax rates to be reduced, attracting other businesses to the region as well as inspiring a whole new generation of startups^4^.

While stories such as the success of BioNTech are often retold and remembered, their success is the exception rather than the rule. The overwhelming majority of startups ultimately fail. One study of 775 startups in Canada that successfully attracted external investment found only 35% were still operating seven years later^5^.

But what determines the success of these ‘lucky few’? When assessing the success factors of startups, especially in the early-stage unproven phase, venture capitalists and other investors offer valuable insights. Three different schools of thought characterise their perspectives: first, *supply-side or product investors*: those who prioritise investing in firms they consider to have novel and superior products and services, investing in companies with intellectual property such as patents and trademarks. Secondly, *demand-side or market-based investors*: those who prioritise investing in areas of highest market interest, such as in hot areas of technology like quantum computing or recurrent or emerging large-scale social and economic challenges such as the decarbonisation of the economy. Thirdly, *talent investors*: those who prioritise the foundation team above the startup’s initial products or what industry or problem it is looking to address.

Investors who adopt the third perspective and prioritise talent often recognise that a good team can overcome many challenges in the lead-up to *product-market* fit. And while the initial products of a startup may or may not work a successful and well-functioning team has the potential to pivot to new markets and new products, even if the initial ones prove untenable. Not surprisingly, an industry ‘autopsy’ into 101 tech startup failures found 23% were due to not having the right team—the number three cause of failure ahead of running out of cash or not having a product that meets the market need^6^.

Accordingly, early entrepreneurship research was focused on the personality of founders, but the focus shifted away in the mid-1980s onwards towards more environmental factors such as venture capital financing^7–9^, networks^10^, location^11^ and due to a range of issues and challenges identified with the early entrepreneurship personality research^12, 13^. At the turn of the 21st century, some scholars began exploring ways to combine context and personality and reconcile entrepreneurs’ individual traits with features of their environment. In her influential work ’The Sociology of Entrepreneurship’, Patricia H. Thornton^14^ discusses two perspectives on entrepreneurship: the supply-side perspective (personality theory) and the demand-side perspective (environmental approach). The supply-side perspective focuses on the individual traits of entrepreneurs. In contrast, the demand-side perspective focuses on the context in which entrepreneurship occurs, with factors such as finance, industry and geography each playing their part. In the past two decades, there has been a revival of interest and research that explores how entrepreneurs’ personality relates to the success of their ventures. This new and growing body of research includes several reviews and meta-studies, which show that personality traits play an important role in both career success and entrepreneurship^15–19^, that there is heterogeneity in definitions and samples used in research on entrepreneurship^16, 18^, and that founder personality plays an important role in overall startup outcomes^17, 19^.

Motivated by the pivotal role of the personality of founders on startup success outlined in these recent contributions, we investigate two main research questions:

1. Which personality features characterise founders?
2. Do their personalities, particularly the diversity of personality types in founder teams, play a role in startup success?

We aim to understand whether certain founder personalities and their combinations relate to startup success, defined as whether their company has been acquired, acquired another company or listed on a public stock exchange. For the quantitative analysis, we draw on a previously published methodology^20^, which matches people to their ‘ideal’ jobs based on social media-inferred personality traits.

We find that personality traits matter for startup success. In addition to firm-level factors of location, industry and company age, we show that founders’ specific Big Five personality traits, such as adventurousness and openness, are significantly more widespread among successful startups. As we find that companies with multi-founder teams are more likely to succeed, we cluster founders in six different and distinct personality groups to underline the relevance of the complementarity in personality traits among founder teams. Startups with diverse and specific combinations of founder types (e. g., an adventurous ‘Leader’, a conscientious ‘Accomplisher’, and an extroverted ‘Developer’) have significantly higher odds of success.

We organise the rest of this paper as follows. In the Section "Results", we introduce the data used and the methods applied to relate founders’ psychological traits with their startups’ success. We introduce the natural language processing method to derive individual and team personality characteristics and the clustering technique to identify personality groups. Then, we present the result for multi-variate regression analysis that allows us to relate firm success with external and personality features. Subsequently, the Section "Discussion" mentions limitations and opportunities for future research in this domain. In the Section "Methods", we describe the data, the variables in use, and the clustering in greater detail. Robustness checks and additional analyses can be found in the Supplementary Information.

## Results

### Data

Our analysis relies on two datasets. We infer individual personality facets via a previously published methodology^20^ from *Twitter* user profiles. Here, we restrict our analysis to founders with a *Crunchbase* profile. Crunchbase is the world’s largest directory on startups. It provides information about more than one million companies, primarily focused on funding and investors. A company’s public Crunchbase profile can be considered a digital business card of an early-stage venture. As such, the founding teams tend to provide information about themselves, including their educational background or a link to their Twitter account.

We infer the personality profiles of the founding teams of early-stage ventures from their publicly available Twitter profiles, using the methodology described by Kern et al.^20^. Then, we correlate this information to data from Crunchbase to determine whether particular combinations of personality traits correspond to the success of early-stage ventures. The final dataset used in the success prediction model contains n = 21,187 startup companies (for more details on the data see the Methods section and SI section A.5).

Revisions of Crunchbase as a data source for investigations on a firm and industry level confirm the platform to be a useful and valuable source of data for startups research, as comparisons with other sources at micro-level, e.g., VentureXpert or PwC, also suggest that the platform’s coverage is very comprehensive, especially for start-ups located in the United States^21^. Moreover, aggregate statistics on funding rounds by country and year are quite similar to those produced with other established sources, going to validate the use of Crunchbase as a reliable source in terms of coverage of funded ventures. For instance, Crunchbase covers about the same number of investment rounds in the analogous sectors as collected by the National Venture Capital Association^22^. However, we acknowledge that the data source might suffer from registration latency (a certain delay between the foundation of the company and its actual registration on Crunchbase) and success bias in company status (the likeliness that failed companies decide to delete their profile from the database).

### The definition of startup success

The success of startups is uncertain, dependent on many factors and can be measured in various ways. Due to the likelihood of failure in startups, some large-scale studies have looked at which features predict startup survival rates^23^, and others focus on fundraising from external investors at various stages^24^. Success for startups can be measured in multiple ways, such as the amount of external investment attracted, the number of new products shipped or the annual growth in revenue. But sometimes external investments are misguided, revenue growth can be short-lived, and new products may fail to find traction.

Success in a startup is typically staged and can appear in different forms and times. For example, a startup may be seen to be successful when it finds a clear solution to a widely recognised problem, such as developing a successful vaccine. On the other hand, it could be achieving some measure of commercial success, such as rapidly accelerating sales or becoming profitable or at least cash positive. Or it could be reaching an exit for foundation investors via a trade sale, acquisition or listing of its shares for sale on a public stock exchange via an Initial Public Offering (IPO).

For our study, we focused on the startup’s extrinsic success rather than the founders’ intrinsic success per se, as its more visible, objective and measurable. A frequently considered measure of success is the attraction of external investment by venture capitalists^25^. However, this is not in and of itself a good measure of clear, incontrovertible success, particularly for early-stage ventures. This is because it reflects investors’ expectations of a startup’s success potential rather than actual business success. Similarly, we considered other measures like revenue growth^26^, liquidity events^27–29^, profitability^30^ and social impact^31^, all of which have benefits as they capture incremental success, but each also comes with operational measurement challenges.

Therefore, we apply the success definition initially introduced by Bonaventura et al.^32^, namely that a startup is acquired, acquires another company or has an initial public offering (IPO). We consider any of these major capital liquidation events as a clear threshold signal that the company has matured from an early-stage venture to becoming or is on its way to becoming a mature company with clear and often significant business growth prospects. Together these three major liquidity events capture the primary forms of exit for external investors (an acquisition or trade sale and an IPO). For companies with a longer autonomous growth runway, acquiring another company marks a similar milestone of scale, maturity and capability.

Using multifactor analysis and a binary classification prediction model of startup success, we looked at many variables together and their relative influence on the probability of the success of startups. We looked at seven categories of factors through three lenses of firm-level factors: (1) location, (2) industry, (3) age of the startup; founder-level factors: (4) number of founders, (5) gender of founders, (6) personality characteristics of founders and; lastly team-level factors: (7) founder-team personality combinations. The model performance and relative impacts on the probability of startup success of each of these categories of founders are illustrated in more detail in section A.6 of the Supplementary Information (in particular Extended Data Fig. 19 and Extended Data Fig. 20). In total, we considered over three hundred variables (n = 323) and their relative significant associations with success.

### The personality of founders

Besides product-market, industry, and firm-level factors (see SI section A.1), research suggests that the personalities of founders play a crucial role in startup success^19^. Therefore, we examine the personality characteristics of individual startup founders and teams of founders in relationship to their firm’s success by applying the success definition used by Bonaventura et al.^32^.

Employing established methods^33–35^, we inferred the personality traits across 30 dimensions (Big Five facets) of a large global sample of startup founders. The startup founders cohort was created from a subset of founders from the global startup industry directory Crunchbase, who are also active on the social media platform Twitter.

To measure the personality of the founders, we used the Big Five, a popular model of personality which includes five core traits: Openness to Experience, Conscientiousness, Extraversion, Agreeableness, and Emotional stability. Each of these traits can be further broken down into thirty distinct facets. Studies have found that the Big Five predict meaningful life outcomes, such as physical and mental health, longevity, social relationships, health-related behaviours, antisocial behaviour, and social contribution, at levels on par with intelligence and socioeconomic status^36^Using machine learning to infer personality traits by analysing the use of language and activity on social media has been shown to be more accurate than predictions of coworkers, friends and family and similar in accuracy to the judgement of spouses^37^. Further, as other research has shown, we assume that personality traits remain stable in adulthood even through significant life events^38–40^. Personality traits have been shown to emerge continuously from those already evident in adolescence^41^ and are not significantly influenced by external life events such as becoming divorced or unemployed^42^. This suggests that the direction of any measurable effect goes from founder personalities to startup success and not vice versa.

**Figure 1:**
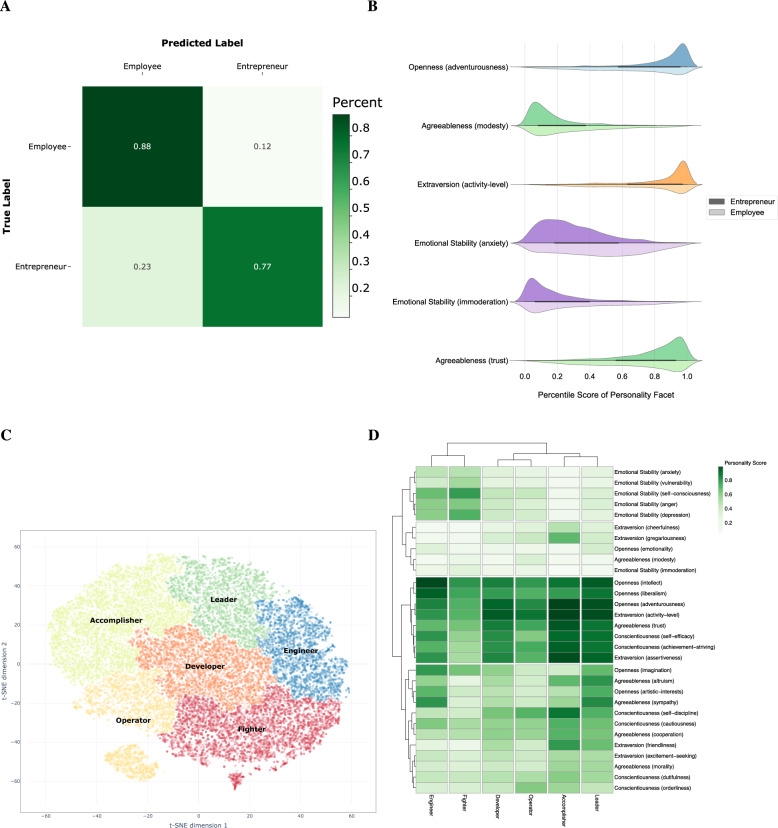
Founder-Level Factors of Startup Success. (**A**), Successful entrepreneurs differ from successful employees. They can be accurately distinguished using a classifier with personality information alone. (**B**), Successful entrepreneurs have different Big Five facet distributions, especially on adventurousness, modesty and activity level. (**C**), Founders come in six different types: Fighters, Operators, Accomplishers, Leaders, Engineers and Developers (FOALED) (**D**), Each founder Personality-Type has its distinct facet.

As a first investigation to what extent personality traits might relate to entrepreneurship, we use the personality characteristics of individuals to predict whether they were an entrepreneur or an employee. We trained and tested a machine-learning random forest classifier to distinguish and classify entrepreneurs from employees and vice-versa using inferred personality vectors alone. As a result, we found we could correctly predict *entrepreneurs* with 77% accuracy and *employees* with 88% accuracy (Fig. 1A). Thus, based on personality information alone, we correctly predict all unseen new samples with 82.5% accuracy (See SI section A.2 for more details on this analysis, the classification modelling and prediction accuracy).

We explored in greater detail which personality features are most prominent among entrepreneurs. We found that the subdomain or facet of *Adventurousness* within the Big Five Domain of Openness was significant and had the largest effect size. The facet of *Modesty* within the Big Five Domain of Agreeableness and *Activity Level* within the Big Five Domain of Extraversion was the subsequent most considerable effect (Fig. 1B). Adventurousness in the Big Five framework is defined as the preference for variety, novelty and starting new things—which are consistent with the role of a startup founder whose role, especially in the early life of the company, is to explore things that do not scale easily^43^ and is about developing and testing new products, services and business models with the market.

Once we derived and tested the Big Five personality features for each entrepreneur in our data set, we examined whether there is evidence indicating that startup founders naturally cluster according to their personality features using a Hopkins test (see Extended Data Figure 6). We discovered clear clustering tendencies in the data compared with other renowned reference data sets known to have clusters. Then, once we established the founder data clusters, we used agglomerative hierarchical clustering. This ‘bottom-up’ clustering technique initially treats each observation as an individual cluster. Then it merges them to create a hierarchy of possible cluster schemes with differing numbers of groups (See Extended Data Fig. 7). And lastly, we identified the optimum number of clusters based on the outcome of four different clustering performance measurements: Davies-Bouldin Index, Silhouette coefficients, Calinski-Harabas Index and Dunn Index (see Extended Data Figure 8). We find that the optimum number of clusters of startup founders based on their personality features is six (labelled #0 through to #5), as shown in Fig. 1C.

To better understand the context of different founder types, we positioned each of the six types of founders within an occupation-personality matrix established from previous research^44^. This research showed that ‘each job has its own personality’ using a substantial sample of employees across various jobs. Utilising the methodology employed in this study, we assigned labels to the cluster names #0 to #5, which correspond to the identified *occupation tribes* that best describe the personality facets represented by the clusters (see Extended Data Fig. 9 for an overview of these tribes, as identified by McCarthy et al.^44^).

**Table 1:** Typology of Founders by Personality. Six different types of founders are revealed by clustering founders (n = 32 k) by their Big Five personality facets.

Founder type		Distinctive Personality Traits
Clustered by personality (Cluster code)		Personality traits of founders in this cluster (Big Five facets)
Fighter	(#5)	Emotional stability (anger, anxiety, depression, immoderation, self-consciousness, vulnerability)
Operator	(#4)	Highest in conscientiousness in the facet of orderliness and high agreeableness in the facet of humility for founders in this cluster.
Accomplisher	(#0)	Highly extraverted (all facets) and conscientious (five facets)
Leader	(#2)	Highest in openness in the facets of artistic interests and emotionality also highest in agreeableness in facets of altruism and sympathy.
Expert/Engineer	(#1)	Highest in openness in the facets of imagination and intellect.
Developer	(#3)	’Middle child’ cluster—no facets are maximums or minimums, but it shares characteristics similar to fighters but higher in extraversion.

Each type—Fighter, Operator, Accomplisher, Leader, Engineer and Developer (FOALED)—has its distinctive personality footprint.

Utilising this approach, we identify three ’purebred’ clusters: #0, #2 and #5, whose members are dominated by a single tribe (larger than 60% of all individuals in each cluster are characterised by one tribe). Thus, these clusters represent and share personality attributes of these previously identified occupation-personality tribes^44^, which have the following known distinctive personality attributes (see also Table 1):

- *Accomplishers (#0)*—Organised & outgoing. confident, down-to-earth, content, accommodating, mild-tempered & self-assured.
- *Leaders (#2)*—Adventurous, persistent, dispassionate, assertive, self-controlled, calm under pressure, philosophical, excitement-seeking & confident.
- *Fighters (#5)*—Spontaneous and impulsive, tough, sceptical, and uncompromising.

We labelled these clusters with the tribe names, acknowledging that labels are somewhat arbitrary, based on our best interpretation of the data (See SI section A.3 for more details).

For the remaining three clusters #1, #3 and #4, we can see they are ‘hybrids’, meaning that the founders within them come from a mix of different tribes, with no one tribe representing more than 50% of the members of that cluster. However, the tribes with the largest share were noted as #1 Experts/Engineers, #3 Fighters, and #4 Operators.

To label these three hybrid clusters, we examined the closest occupations to the median personality features of each cluster. We selected a name that reflected the common themes of these occupations, namely:

- *Experts/Engineers (#1)* as the closest roles included Materials Engineers and Chemical Engineers. This is consistent with this cluster’s personality footprint, which is highest in openness in the facets of imagination and intellect.
- *Developers (#3)* as the closest roles include Application Developers and related technology roles such as Business Systems Analysts and Product Managers.
- *Operators (#4)* as the closest roles include service, maintenance and operations functions, including Bicycle Mechanic, Mechanic and Service Manager. This is also consistent with one of the key personality traits of high conscientiousness in the facet of orderliness and high agreeableness in the facet of humility for founders in this cluster.

Together, these six different types of startup founders (Fig. 1C) represent a framework we call the FOALED model of founder types—an acronym of Fighters, Operators, Accomplishers, Leaders, Engineers and **D**evelopers.

Each founder’s personality type has its distinct facet footprint (for more details, see Extended Data Figure 10 in SI section A.3). Also, we observe a central core of correlated features that are high for all types of entrepreneurs, including intellect, adventurousness and activity level (Fig. 1D).To test the robustness of the clustering of the personality facets, we compare the mean scores of the individual facets per cluster with a 20-fold resampling of the data and find that the clusters are, overall, largely robust against resampling (see Extended Data Figure 11 in SI section A.3 for more details).

We also find that the clusters accord with the distribution of founders’ roles in their startups. For example, Accomplishers are often Chief Executive Officers, Chief Financial Officers, or Chief Operating Officers, while Fighters tend to be Chief Technical Officers, Chief Product Officers, or Chief Commercial Officers (see Extended Data Fig. 12 in SI section A.4 for more details).

### The ensemble theory of success

**Figure 2:**
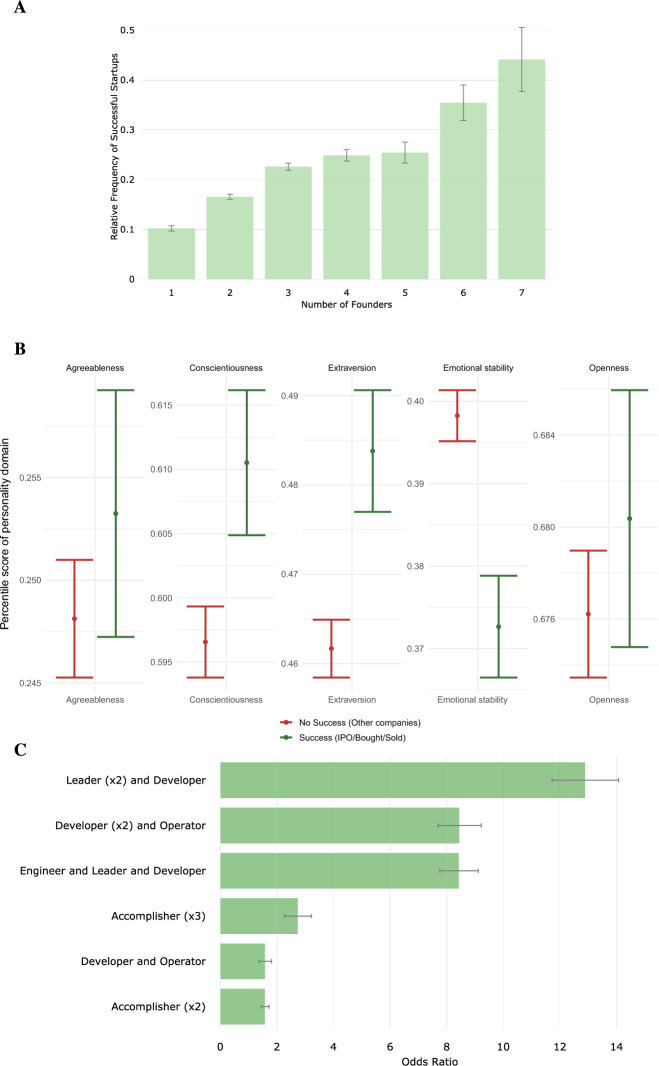
The Ensemble Theory of Team-Level Factors of Startup Success. (**A**) Having a larger founder team elevates the chances of success. This can be due to multiple reasons, e.g., a more extensive network or knowledge base but also personality diversity. (**B**) We show that joint personality combinations of founders are significantly related to higher chances of success. This is because it takes more than one founder to cover all beneficial personality traits that ‘breed’ success. (**C**) In our multifactor model, we show that firms with diverse and specific combinations of types of founders have significantly higher odds of success.

While founders’ individual personality traits, such as Adventurousness or Openness, show to be related to their firms’ success, we also hypothesise that the combination, or ensemble, of personality characteristics of a founding team impacts the chances of success. The logic behind this reasoning is complementarity, which is proposed by contemporary research on the functional roles of founder teams. Examples of these clear functional roles have evolved in established industries such as film and television, construction, and advertising^45^. When we subsequently explored the combinations of personality types among founders and their relationship to the probability of startup success, adjusted for a range of other factors in a multi-factorial analysis, we found significantly increased chances of success for mixed foundation teams:

Initially, we find that firms with multiple founders are more likely to succeed, as illustrated in Fig. 2A, which shows firms with three or more founders are more than twice as likely to succeed than solo-founded startups. This finding is consistent with investors’ advice to founders and previous studies^46^. We also noted that some personality types of founders increase the probability of success more than others, as shown in SI section A.6 (Extended Data Figures 16 and 17). Also, we note that gender differences play out in the distribution of personality facets: successful female founders and successful male founders show facet scores that are more similar to each other than are non-successful female founders to non-successful male founders (see Extended Data Figure 18).

Access to more extensive networks and capital could explain the benefits of having more founders. Still, as we find here, it also offers a greater diversity of combined personalities, naturally providing a broader range of maximum traits. So, for example, one founder may be more open and adventurous, and another could be highly agreeable and trustworthy, thus, potentially complementing each other’s particular strengths associated with startup success.

The benefits of larger and more personality-diverse foundation teams can be seen in the apparent differences between successful and unsuccessful firms based on their combined Big Five personality team footprints, as illustrated in Fig. 2B. Here, maximum values for each Big Five trait of a startup’s co-founders are mapped; stratified by successful and non-successful companies. Founder teams of successful startups tend to score higher on Openness, Conscientiousness, Extraversion, and Agreeableness.

When examining the combinations of founders with different personality types, we find that some ensembles of personalities were significantly correlated with greater chances of startup success—while controlling for other variables in the model—as shown in Fig. 2C (for more details on the modelling, the predictive performance and the coefficient estimates of the final model, see Extended Data Figures 19, 20, and 21 in SI section A.6).

Three combinations of trio-founder companies were more than twice as likely to succeed than other combinations, namely teams with (1) a *Leader* and two *Developers*, (2) an *Operator* and two *Developers*, and (3) an *Expert/Engineer*, *Leader* and *Developer*. To illustrate the potential mechanisms on how personality traits might influence the success of startups, we provide some examples of well-known, successful startup founders and their characteristic personality traits in Extended Data Figure 22.

## Discussion

Startups are one of the key mechanisms for brilliant ideas to become solutions to some of the world’s most challenging economic and social problems. Examples include the Google search algorithm, disability technology startup Fingerwork’s touchscreen technology that became the basis of the Apple iPhone, or the Biontech mRNA technology that powered Pfizer’s COVID-19 vaccine.

We have shown that founders’ personalities and the combination of personalities in the founding team of a startup have a material and significant impact on its likelihood of success. We have also shown that successful startup founders’ personality traits are significantly different from those of successful employees—so much so that a simple predictor can be trained to distinguish between employees and entrepreneurs with more than 80% accuracy using personality trait data alone.

Just as *occupation-personality* maps derived from data can provide career guidance tools, so too can data on successful entrepreneurs’ personality traits help people decide whether becoming a founder may be a good choice for them.

We have learnt through this research that there is not one type of ideal ’entrepreneurial’ personality but six different types. Many successful startups have multiple co-founders with a combination of these different personality types.

To a large extent, founding a startup is a team sport; therefore, diversity and complementarity of personalities matter in the foundation team. It has an outsized impact on the company’s likelihood of success. While all startups are high risk, the risk becomes lower with more founders, particularly if they have distinct personality traits.

Our work demonstrates the benefits of *personality diversity* among the founding team of startups. Greater awareness of this novel form of diversity may help create more resilient startups capable of more significant innovation and impact.

The data-driven research approach presented here comes with certain methodological limitations. The principal data sources of this study—Crunchbase and Twitter—are extensive and comprehensive, but there are characterised by some known and likely sample biases.

Crunchbase is the principal public chronicle of venture capital funding. So, there is some likely sample bias toward: (1) Startup companies that are funded externally: self-funded or bootstrapped companies are less likely to be represented in Crunchbase; (2) technology companies, as that is Crunchbase’s roots; (3) multi-founder companies; (4) male founders: while the representation of female founders is now double that of the mid-2000s, women still represent less than 25% of the sample; (5) companies that succeed: companies that fail, especially those that fail early, are likely to be less represented in the data.

Samples were also limited to those founders who are active on Twitter, which adds additional selection biases. For example, Twitter users typically are younger, more educated and have a higher median income^47^. Another limitation of our approach is the potentially biased presentation of a person’s digital identity on social media, which is the basis for identifying personality traits. For example, recent research suggests that the language and emotional tone used by entrepreneurs in social media can be affected by events such as business failure^48^, which might complicate the personality trait inference.

In addition to sampling biases within the data, there are also significant historical biases in startup culture. For many aspects of the entrepreneurship ecosystem, women, for example, are at a disadvantage^49^. Male-founded companies have historically dominated most startup ecosystems worldwide, representing the majority of founders and the overwhelming majority of venture capital investors. As a result, startups with women have historically attracted significantly fewer funds^50^, in part due to the male bias among venture investors, although this is now changing, albeit slowly^51^.

The research presented here provides quantitative evidence for the relevance of personality types and the diversity of personalities in startups. At the same time, it brings up other questions on how personality traits are related to other factors associated with success, such as:

1. Will the recent growing focus on promoting and investing in female founders change the nature, composition and dynamics of startups *and* their personalities leading to a more diverse personality landscape in startups?
2. Will the growth of startups outside of the United States change what success looks like to investors and hence the role of different personality traits and their association to diverse success metrics?
3. Many of today’s most renowned entrepreneurs are either *Baby Boomers* (such as Gates, Branson, Bloomberg) or *Generation Xers* (such as Benioff, Cannon-Brookes, Musk). However, as we can see, personality is both a predictor and driver of success in entrepreneurship. Will generation-wide differences in personality and outlook affect startups and their success?

Moreover, the findings shown here have natural extensions and applications beyond startups, such as for new projects within large established companies. While not technically startups, many large enterprises and industries such as construction, engineering and the film industry rely on forming new project-based, cross-functional teams that are often new ventures and share many characteristics of startups.

There is also potential for extending this research in other settings in government, NGOs, and within the research community. In scientific research, for example, team diversity in terms of age, ethnicity and gender has been shown to be predictive of impact, and personality diversity may be another critical dimension^52^.

Another extension of the study could investigate the development of the language used by startup founders on social media over time. Such an extension could investigate whether the language (and inferred psychological characteristics) change as the entrepreneurs’ ventures go through major business events such as foundation, funding, or exit.

Overall, this study demonstrates, first, that startup founders have significantly different personalities than employees. Secondly, besides *firm-level* factors, which are known to influence firm success, we show that a range of *founder-level* factors, notably the character traits of its founders, significantly impact a startup’s likelihood of success. Lastly, we looked at *team-level* factors. We discovered in a multifactor analysis that personality-diverse teams have the most considerable impact on the probability of a startup’s success, underlining the importance of personality diversity as a relevant factor of team performance and success.

## Methods

### Data sources

#### Entrepreneurs dataset

Data about the founders of startups were collected from Crunchbase (Table 2), an open reference platform for business information about private and public companies, primarily early-stage startups. It is one of the largest and most comprehensive data sets of its kind and has been used in over 100 peer-reviewed research articles about economic and managerial research.

Crunchbase contains data on over two million companies - mainly startup companies and the companies who partner with them, acquire them and invest in them, as well as profiles on well over one million individuals active in the entrepreneurial ecosystem worldwide from over 200 countries and spans. Crunchbase started in the technology startup space, and it now covers all sectors, specifically focusing on entrepreneurship, investment and high-growth companies.

While Crunchbase contains data on over one million individuals in the entrepreneurial ecosystem, some are not entrepreneurs or startup founders but play other roles, such as investors, lawyers or executives at companies that acquire startups. To create a subset of only entrepreneurs, we selected a subset of 32,732 who self-identify as founders and co-founders (by job title) and who are also publicly active on the social media platform Twitter. We also removed those who also are venture capitalists to distinguish between investors and founders.

We selected founders active on Twitter to be able to use natural language processing to infer their Big Five personality features using an open-vocabulary approach shown to be accurate in the previous research by analysing users’ unstructured text, such as Twitter posts in our case. For this project, as with previous research^20^, we employed a commercial service, IBM Watson Personality Insight, to infer personality facets. This service provides raw scores and percentile scores of Big Five Domains (Openness, Conscientiousness, Extraversion, Agreeableness and Emotional Stability) and the corresponding 30 subdomains or facets. In addition, the public content of Twitter posts was collected, and there are 32,732 profiles that each had enough Twitter posts (more than 150 words) to get relatively accurate personality scores (less than 12.7% Average Mean Absolute Error).

The entrepreneurs’ dataset is analysed in combination with other data about the companies they founded to explore questions about the nature and patterns of personality traits of entrepreneurs and the relationships between these patterns and company success.

For the multifactor analysis, we further filtered the data in several preparatory steps for the success prediction modelling (for more details, see SI section A.5). In particular, we removed data points with missing values (Extended Data Fig. 13) and kept only companies in the data that were founded from 1990 onward to ensure consistency with previous research^32^ (see Extended Data Fig. 14). After cleaning, filtering and pre-processing the data, we ended up with data from 25,214 founders who founded 21,187 startup companies to be used in the multifactor analysis. Of those, 3442 startups in the data were successful, 2362 in the first seven years after they were founded (see Extended Data Figure 15 for more details).

**Table 2:** Summary of the basic information of the entrepreneurs’ dataset the number of founders and associated startups in the study population, how many countries those startups are across, and the time span the data collected covers, the number of features included).

Founders with personality data	Associated startups	Countries	Date range	Founders individual features
32,732	23,292	215	2008-2021	100

#### Entrepreneurs and employees dataset

To investigate whether startup founders show personality traits that are similar or different from the population at large (i. e. the entrepreneurs vs employees sub-analysis shown in Fig. 1A and B), we filtered the entrepreneurs’ data further: we reduced the sample to those founders of companies, which attracted more than US$100k in investment to create a reference set of successful entrepreneurs (n$=$4400).

To create a control group of employees who are *not also entrepreneurs* or very unlikely to be of have been entrepreneurs, we leveraged the fact that while some occupational titles like CEO, CTO and *Public Speaker* are commonly shared by founders and co-founders, some others such as *Cashier*, *Zoologist* and *Detective* very rarely co-occur seem to be founders or co-founders. To illustrate, many company founders also adopt regular occupation titles such as CEO or CTO. Many founders will be Founder and CEO or Co-founder and CTO. While founders are often CEOs or CTOs, the reverse is not necessarily true, as many CEOs are professional executives that were not involved in the establishment or ownership of the firm.

Using data from LinkedIn, we created an *Entrepreneurial Occupation Index (EOI)* based on the ratio of entrepreneurs for each of the 624 occupations used in a previous study of occupation-personality fit^44^. It was calculated based on the percentage of all people working in the occupation from LinkedIn compared to those who shared the title Founder or Co-founder (See SI section A.2 for more details). A reference set of employees (n=6685) was then selected across the 112 different occupations with the lowest propensity for entrepreneurship (less than 0.5% EOI) from a large corpus of Twitter users with known occupations, which is also drawn from the previous occupational-personality fit study^44^.

These two data sets were used to test whether it may be possible to distinguish successful entrepreneurs from successful employees based on the different patterns of personality traits alone.

### Hierarchical clustering

We applied several clustering techniques and tests to the personality vectors of the entrepreneurs’ data set to determine if there are natural clusters and, if so, how many are the optimum number.

Firstly, to determine if there is a natural typology to founder personalities, we applied the Hopkins statistic—a statistical test we used to answer whether the entrepreneurs’ dataset contains inherent clusters. It measures the clustering tendency based on the ratio of the sum of distances of real points within a sample of the entrepreneurs’ dataset to their nearest neighbours and the sum of distances of randomly selected artificial points from a simulated uniform distribution to their nearest neighbours in the real entrepreneurs’ dataset. The ratio measures the difference between the entrepreneurs’ data distribution and the simulated uniform distribution, which tests the randomness of the data. The range of Hopkins statistics is from 0 to 1. The scores are close to 0, 0.5 and 1, respectively, indicating whether the dataset is uniformly distributed, randomly distributed or highly clustered.

To cluster the founders by personality facets, we used Agglomerative Hierarchical Clustering (AHC)—a bottom-up approach that treats an individual data point as a singleton cluster and then iteratively merges pairs of clusters until all data points are included in the single big collection. Ward’s linkage method is used to choose the pair of groups for minimising the increase in the within-cluster variance after combining. AHC was widely applied to clustering analysis since a tree hierarchy output is more informative and interpretable than K-means. Dendrograms were used to visualise the hierarchy to provide the perspective of the optimal number of clusters. The heights of the dendrogram represent the distance between groups, with lower heights representing more similar groups of observations. A horizontal line through the dendrogram was drawn to distinguish the number of significantly different clusters with higher heights. However, as it is not possible to determine the optimum number of clusters from the dendrogram, we applied other clustering performance metrics to analyse the optimal number of groups.

A range of Clustering performance metrics were used to help determine the optimal number of clusters in the dataset after an apparent clustering tendency was confirmed. The following metrics were implemented to evaluate the differences between within-cluster and between-cluster distances comprehensively: Dunn Index, Calinski-Harabasz Index, Davies-Bouldin Index and Silhouette Index. The Dunn Index measures the ratio of the minimum inter-cluster separation and the maximum intra-cluster diameter. At the same time, the Calinski-Harabasz Index improves the measurement of the Dunn Index by calculating the ratio of the average sum of squared dispersion of inter-cluster and intra-cluster. The Davies-Bouldin Index simplifies the process by treating each cluster individually. It compares the sum of the average distance among intra-cluster data points to the cluster centre of two separate groups with the distance between their centre points. Finally, the Silhouette Index is the overall average of the silhouette coefficients for each sample. The coefficient measures the similarity of the data point to its cluster compared with the other groups. Higher scores of the Dunn, Calinski-Harabasz and Silhouette Index and a lower score of the Davies-Bouldin Index indicate better clustering configuration.

### Classification modelling

#### Classification algorithms

To obtain a comprehensive and robust conclusion in the analysis predicting whether a given set of personality traits corresponds to an entrepreneur or an employee, we explored the following classifiers: Naïve Bayes, Elastic Net regularisation, Support Vector Machine, Random Forest, Gradient Boosting and Stacked Ensemble. The Naïve Bayes classifier is a probabilistic algorithm based on Bayes’ theorem with assumptions of independent features and equiprobable classes. Compared with other more complex classifiers, it saves computing time for large datasets and performs better if the assumptions hold. However, in the real world, those assumptions are generally violated. Elastic Net regularisation combines the penalties of Lasso and Ridge to regularise the Logistic classifier. It eliminates the limitation of multicollinearity in the Lasso method and improves the limitation of feature selection in the Ridge method. Even though Elastic Net is as simple as the Naïve Bayes classifier, it is more time-consuming. The Support Vector Machine (SVM) aims to find the ideal line or hyperplane to separate successful entrepreneurs and employees in this study. The dividing line can be non-linear based on a non-linear kernel, such as the Radial Basis Function Kernel. Therefore, it performs well on high-dimensional data while the ’right’ kernel selection needs to be tuned. Random Forest (RF) and Gradient Boosting Trees (GBT) are ensembles of decision trees. All trees are trained independently and simultaneously in RF, while a new tree is trained each time and corrected by previously trained trees in GBT. RF is a more robust and straightforward model since it does not have many hyperparameters to tune. GBT optimises the objective function and learns a more accurate model since there is a successive learning and correction process. Stacked Ensemble combines all existing classifiers through a Logistic Regression. Better than bagging with only variance reduction and boosting with only bias reduction, the ensemble leverages the benefit of model diversity with both lower variance and bias. All the above classification algorithms distinguish successful entrepreneurs and employees based on the personality matrix.

#### Evaluation metrics

A range of evaluation metrics comprehensively explains the performance of a classification prediction. The most straightforward metric is accuracy, which measures the overall portion of correct predictions. It will mislead the performance of an imbalanced dataset. The F1 score is better than accuracy by combining precision and recall and considering the False Negatives and False Positives. Specificity measures the proportion of detecting the true negative rate that correctly identifies employees, while Positive Predictive Value (PPV) calculates the probability of accurately predicting successful entrepreneurs. Area Under the Receiver Operating Characteristic Curve (AUROC) determines the capability of the algorithm to distinguish between successful entrepreneurs and employees. A higher value means the classifier performs better on separating the classes.

#### Feature importance

To further understand and interpret the classifier, it is critical to identify variables with significant predictive power on the target. Feature importance of tree-based models measures Gini importance scores for all predictors, which evaluate the overall impact of the model after cutting off the specific feature. The measurements consider all interactions among features. However, it does not provide insights into the directions of impacts since the importance only indicates the ability to distinguish different classes.

#### Statistical analysis

T-test, Cohen’s D and two-sample Kolmogorov-Smirnov test are introduced to explore how the mean values and distributions of personality facets between entrepreneurs and employees differ. The T-test is applied to determine whether the mean of personality facets of two group samples are significantly different from one another or not. The facets with significant differences detected by the hypothesis testing are critical to separate the two groups. Cohen’s d is to measure the effect size of the results of the previous t-test, which is the ratio of the mean difference to the pooled standard deviation. A larger Cohen’s d score indicates that the mean difference is greater than the variability of the whole sample. Moreover, it is interesting to check whether the two groups’ personality facets’ probability distributions are from the same distribution through the two-sample Kolmogorov-Smirnov test. There is no assumption about the distributions, but the test is sensitive to deviations near the centre rather than the tail.

### Privacy and ethics

The focus of this research is to provide high-level insights about groups of startups, founders and types of founder teams rather than on specific individuals or companies. While we used unit record data from the publicly available data of company profiles from *Crunchbase*, we removed all identifiers from the underlying data on individual companies and founders and generated aggregate results, which formed the basis for our analysis and conclusions.

### Supplementary Information

Supplementary Information.

## Data Availability

A dataset which includes only aggregated statistics about the success of startups and the factors that influence is released as part of this research. Underlying data for all figures and the code to reproduce them are available on GitHub: https://github.com/Braesemann/FounderPersonalities. Please contact Fabian Braesemann (fabian.braesemann@oii.ox.ac.uk) in case you have any further questions.
