# Author Correction: The impact of founder personalities on startup success

**DOI:** 10.1038/s41598-024-61082-7

**Published:** 2024-05-07

**Authors:** Paul X. McCarthy, Xian Gong, Fabian Braesemann, Fabian Stephany, Marian-Andrei Rizoiu, Margaret L. Kern

**Affiliations:** 1 The Data Science Institute, University of Technology Sydney, Sydney, NSW Australia; 2 School of Computer Science and Engineering, UNSW Sydney, Sydney, NSW Australia; 3 Faculty of Engineering and Information Technology, University of Technology Sydney, Sydney, Australia; 4 Oxford Internet Institute, University of Oxford, Oxford, UK; 5 DWG Datenwissenschaftliche Gesellschaft Berlin, Berlin, Germany; 6 Melbourne Graduate School of Education, The University of Melbourne, Parkville, VIC Australia

Correction to: *Scientific Reports* 10.1038/s41598-023-41980-y, published online 17 October 2023

The Data Availability section in the original version of this Article was incomplete, the link to the GitHub repository was omitted.

“A dataset which includes only aggregated statistics about the success of startups and the factors that influence is released as part of this research. Underlying data for all figures and the code to reproduce them are also available.

Please contact Fabian Braesemann (fabian.braesemann@oii.ox.ac.uk) in case you require access to any data and code.”

now reads:

“A dataset which includes only aggregated statistics about the success of startups and the factors that influence is released as part of this research. Underlying data for all figures and the code to reproduce them are available on GitHub: https://github.com/Braesemann/FounderPersonalities

Please contact Fabian Braesemann (fabian.braesemann@oii.ox.ac.uk) in case you have any further questions.”

The original Article has been corrected.

