# Supplementary material for: The impact of founder personalities on startup success
Source: Sci Rep. 2023 Oct 17;13:17200. doi: 10.1038/s41598-023-41980-y (PMC10582098; doi:10.1038/s41598-023-41980-y)
Supplement: Supplementary file 1 — Supplementary Information. [file 41598_2023_41980_MOESM1_ESM.pdf]

A Supplementary Information

A.1 Industry- and market-based drivers of startups success

The first lens we looked through was at the *firm-level*. Much of the previous literature on startups has been focused on firm-level or external factors and their influence on success[?]. Startup success has been shown to relate to how much capital the startup has raised, how old it is and what industry it is in, among other things[?].

We, therefore, firstly examine a range of *firm-level* determinants of startup success, including *location* (Extended Data Fig. 1A), *industry* (Extended Data Fig. 1B) and *age of the startup* (Extended Data Fig. 1C) to explore to what extent these factors are associated with success. We find that startup success is influenced strongly by its location (firms from Japan, Scandinavia, USA, France, and Germany are more likely to be successful than those from Turkey, Argentina, Mexico or other countries); industry (firms in Payment Systems and Privacy & Security are most successful) and a company's age.

These findings are used in the multifactor analysis shown in more detail below. The predictive capability of the models that also include personality trait information is compared to those that use only firm-level information.

A.2 Entrepreneurs versus Employees

In addition to our main analysis, we compare the personality traits of entrepreneurs with a reference population of employees. We developed an *Entrepreneurial Occupational Index (EOI; see Extended Data Fig. 2)* based on LinkedIn data that looks at the percentage of people currently employed in that role worldwide and who also hold or have previously held the position of founder or co-founder. We found EOI values for each of the 624 Occupations we have personality profiles for. We ranked the occupations from most entrepreneurial (public speaker 21.11%, chief technology officer 20.75%, and creative director 19.33%) to least entrepreneurial (cashier 0.02%, palaeontologist 0.00%, furniture removalist 0.00%, aged carer 0.00%, bacteriologist 0.00%).

We then created a list of low EOI occupations (n=112), each of which had less than 0.5% of individuals who also held the titles founder or co-founders in their *LinkedIn* Profile. People in these roles may still be founders and co-founders, but it is unlikely that they are. Any individual in even the most entrepreneurial of these 112 occupations (internal auditor) is still five times less likely also to be a founder or co-founder than the global average (2.5%) across all 624 occupations. From our previous study[?], we randomly selected a sample of employees (n=6k) for whom we have inferred personality data and who are unlikely to be entrepreneurs as they are drawn from the 112 low EOI occupations. These employees are Twitter users selected as the first twenty-five search-ranked Twitter users with their occupation specified as part of their biotext. The occupations were selected from standard job titles (O*NET and ANZSCO) and represent a wide cross-section of jobs at differing skill levels and in different industries. De-identified data on employees and their occupations can be requested from the authors.

Using the two samples together (entrepreneurs and employees who are unlikely to be founders), we trained and tested a machine-learning random forest classifier to distinguish and classify entrepreneurs from employees and vice-versa using inferred personality vectors alone. As a result, we could correctly predict entrepreneurs with 77% accuracy and employees with 88% accuracy (Fig. 1A in the main text). Thus, based on personality information alone, we correctly predict all unseen new samples with 82.5% accuracy (See Extended Data Fig. 3 for details on modelling and prediction accuracy).

We explored in greater detail which personality features are most prominent among successful entrepreneurs. We found that the subdomain or facet of *Adventurousness* within the Big Five Domain of Openness was significant and had the largest effect size. This is consistent with previous research that found higher values in the personality trait Openness significantly predict VC financing even after accounting for observable founder and firm characteristics[?] and openness to be the key Big Five Domain that distinguishes entrepreneurs from non-entrepreneurs[?]. The facet of *Modesty* within the Big Five Domain of Agreeableness and *Activity Level* within the Big Five Domain of Extraversion was the subsequent most considerable effect (Fig. 1B in the main text). All thirty dimensions of the Big Five facet were found to be significantly different in their distribution, with ten features having large effect sizes. (See Extended Data Figure 4 for more details on Cohen's D analysis with a complete list of features and their effect sizes, and Extended Data Fig. 5 for Big5 personality facets of employees and entrepreneurs visualised as a heatmap and dendrogram).

Adventurousness in the Big Five framework is defined as the preference for variety, novelty and starting new things - which are consistent with the role of a startup founder whose role, especially in the early life of the company, is to explore things that do not scale easily[?] and is about developing and testing new products, services and business models with the market.

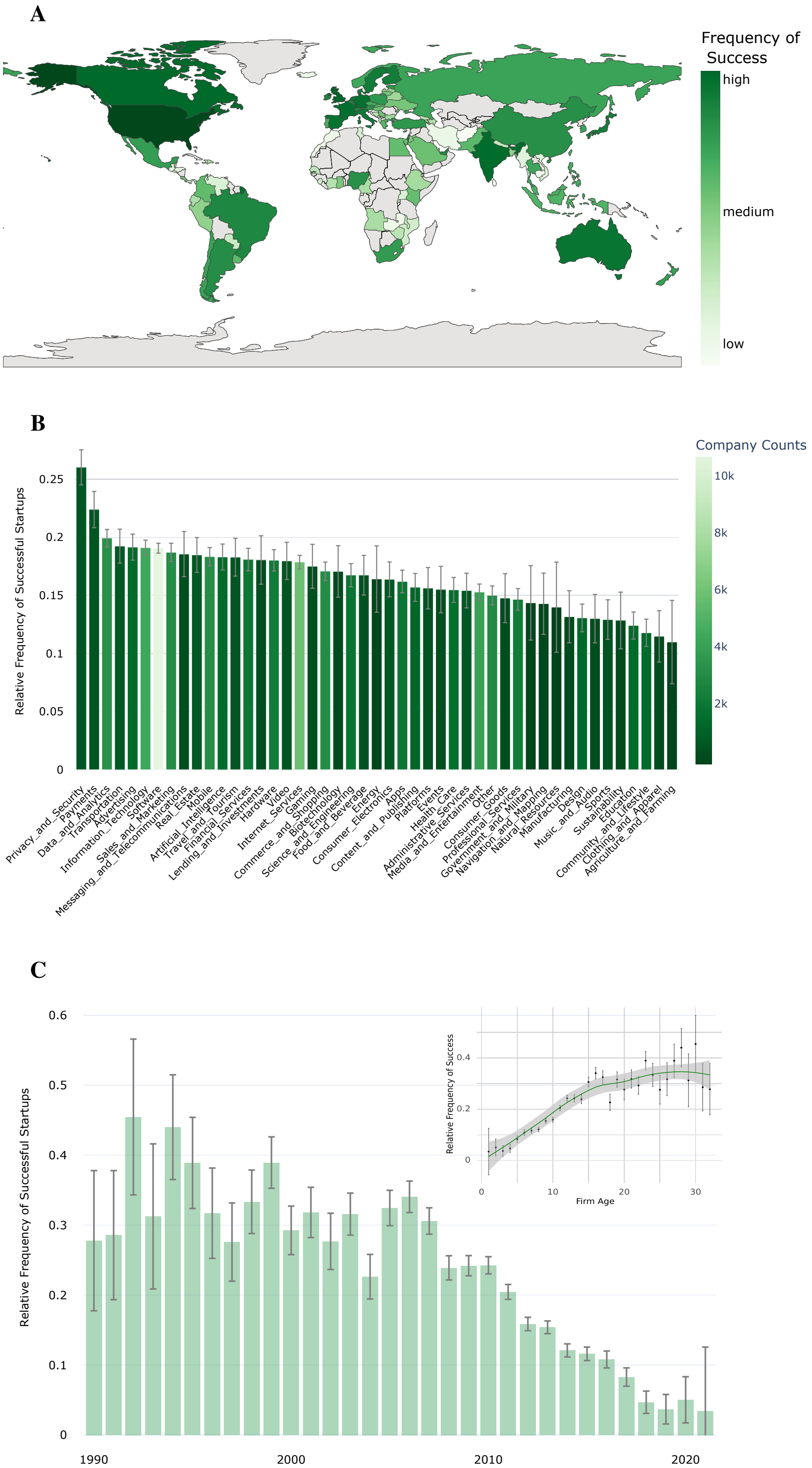

Extended Data Fig. 1. | **Firm-Level Factors of Startup Success. A**, On a country level, chances for success are highest in the US, Japan, West Europe, and Scandinavian countries. **B**, Firms from the payment and software industries have high chances of success. **C**, Chances of success are positively related to a firm's maturity, with firms that are seven years or older having higher chances of success.

Entrepeneurial Occupations Index				
Frequency of people by Occupation				
Occupations (n=624)		Global	Global	Global
**Occupation**		**Count (LinkedIn)**	**with founder or cofounder**	**Index**
PUBLIC SPEAKER	Listeners	18,000	3,800	21.11%
CHIEF TECHNOLOGY OFFICER	Leaders	106,000	22,000	20.75%
CREATIVE DIRECTOR	Listeners	388,000	75,000	19.33%
EXECUTIVE PRODUCER	Listeners	83,000	14,000	16.87%
ARTISTIC DIRECTOR	Rebels	45,000	7,300	16.22%
COLUMNIST	Listeners	27,000	3,900	14.44%
ECONOMIC HISTORIAN	Leaders	21	3	14.29%
CHIEF EXECUTIVE	Leaders	1,080,000	149,000	13.80%
AUTHOR	Rebels	273,000	37,000	13.55%
CHOCOLATE MAKER	Listeners	488	66	13.52%
COMMUNITY ARTIST	Rebels	3,300	439	13.30%
MOTIVATIONAL SPEAKER	Leaders	24,000	3,100	12.92%
EXECUTIVE DIRECTOR	Leaders	924,000	108,000	11.69%
CHIEF OPERATING OFFICER	Accomplishers	209,000	23,000	11.00%
PRODUCT DESIGNER	Listeners	101,000	11,000	10.89%
FILM PRODUCER	Rebels	22,000	2,300	10.45%
MEDIA PRODUCER	Listeners	28,000	2,900	10.36%
ORGANISATIONAL PSYCHOLOGIST	Leaders	890	92	10.34%
MARKETING CONSULTANT	Accomplishers	254,000	26,000	10.24%
PLAYWRIGHT	Rebels	6,800	688	10.12%
BOOK EDITOR	Rebels	6,900	671	9.72%
FILM DIRECTOR	Rebels	38,000	3,500	9.21%
MAGAZINE EDITOR	Listeners	10,000	898	8.98%
SPORT PSYCHOLOGIST	Leaders	1,100	96	8.73%
MANAGING DIRECTOR	Leaders	1,990,000	170,000	8.54%
VLOGGER	Observers	4,100	350	8.54%
ART DIRECTOR	Listeners	201,000	17,000	8.46%
CRITIC	Rebels	8,400	689	8.20%
INSTRUMENTALIST	Observers	2,500	205	8.20%

Low EOI Roles				
Less than 0.5% Global EOI index & > 100 count in Sample				
(Note all Occupation Global EOI = 2.5%)				
Occupations (n=112)		Global	Global	In Sample
**Occupation**		**Count (LinkedIn)**	**Index**	**Count (LinkedIn)**
INTERNAL AUDITOR	Leaders	117,000	0.50%	945
UPHOLSTERER	Fighters	4,700	0.49%	239
PETROLEUM ENGINEER	Fighters	18,000	0.49%	249
PROGRAMMER ANALYST	Fighters	122,000	0.49%	3,100
ANALYST PROGRAMMER	Fighters	122,000	0.49%	3200
SALESPERSON	Fighters	371,000	0.49%	9,800
DETECTIVE	Listeners	51,000	0.48%	1,100
MERCHANDISER	Umpires	313,000	0.48%	4,500
CONTRACT ADMINISTRATOR	Accomplishers	38,000	0.48%	5,400
PARK RANGER	Listeners	8,800	0.48%	363
NAIL TECHNICIAN	Observers	17,000	0.48%	436
RETAIL STORE MANAGER	Accomplishers	51,000	0.47%	2,600
REFERENCE LIBRARIAN	Listeners	8,200	0.46%	137
BOOKKEEPER	Accomplishers	261,000	0.46%	13,000
CONVEYANCER	Accomplishers	7,400	0.46%	2,200
BARISTA	Accomplishers	286,000	0.45%	10,000
CHILD CARE WORKER	Observers	4,800	0.44%	539
COURIER	Accomplishers	67,000	0.44%	2,500
VETERINARY ASSISTANT	Observers	31,000	0.44%	244
DENTAL TECHNICIAN	Fighters	19,000	0.43%	737
CABIN CREW	Umpires	55,000	0.42%	1,500
FLIGHT NURSE	Experts	4,500	0.42%	187
ZOOLOGIST	Rebels	1,200	0.42%	184
BUILDING SURVEYOR	Fighters	13,000	0.42%	1,300
NURSE MANAGER	Leaders	94,000	0.41%	6,700
PURCHASING OFFICER	Accomplishers	25,000	0.40%	3,500
AMBULANCE PARAMEDIC	Leaders	1,000	0.40%	352
ELECTRICAL CONTRACTOR	Accomplishers	17,000	0.40%	1,700

Extended Data Fig. 2. | **Entrepreneurial Occupations Index** To identify a group of individuals who are unlikely to be founders, we rank occupations by the share of founders based on LinkedIn data (see McCarthy et al.[?]); occupations with a share of founders smaller than 0.5 % are assumed to represent individuals unlikely to be entrepreneurs, that is, they are considered as representing 'employee' personalities.

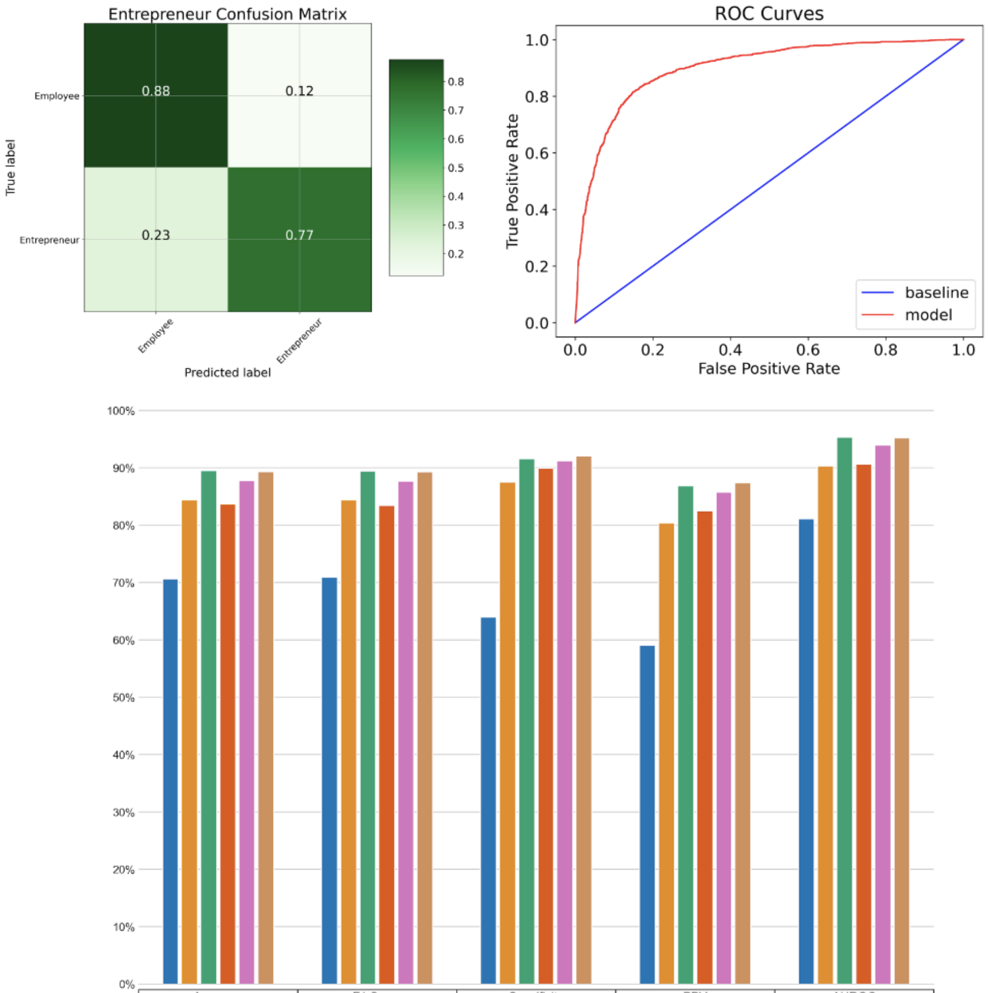

	Accuracy	F1 Score	Specificity	PPV	AUROC
Naive Bayes	71%	71%	64%	59%	81%
Elastic Net	84%	84%	88%	80%	90%
SVM	89%	89%	92%	87%	95%
Random Forest	84%	83%	90%	82%	91%
Gradient Boosting	88%	88%	91%	86%	94%
Stacked Ensemble	89%	89%	92%	87%	95%

Extended Data Fig. 3. | **Entrepreneurial Prediction Performance** The ROC score of the train set is 0.94, while the score of the test set is 0.9. The confusion matrix also demonstrates high accuracy. On the test set, the model could 88% correctly predict the entrepreneurs and 77% correctly predict the employees.

Big 5 Personality Facets	Cohen's D	Effect Size	p Value
Openness (adventurousness)	0.92	Large	0.00
Agreeableness (modesty)	-0.79	Large	0.00
Extraversion (activity_level)	0.78	Large	0.00
Emotional range (anxiety)	-0.77	Large	0.00
Emotional range (immoderation)	-0.73	Large	0.00
Agreeableness (trust)	0.72	Large	0.00
Emotional range (anger)	-0.68	Large	0.00
Emotional range (depression)	-0.68	Large	0.00
Agreeableness (cooperation)	0.67	Large	0.00
Openness (emotionality)	-0.67	Large	0.00
Emotional range (vulnerability)	-0.63	Medium	0.00
Conscientiousness (achievement_striving)	0.59	Medium	0.00
Conscientiousness (self_discipline)	0.55	Medium	0.00
Conscientiousness (cautiousness)	0.50	Medium	0.00
Openness (intellect)	0.45	Medium	0.00
Conscientiousness (self_efficacy)	0.43	Medium	0.00
Extraversion (assertiveness)	0.43	Medium	0.00
Openness (liberalism)	0.43	Medium	0.00
Agreeableness (sympathy)	-0.43	Medium	0.00
Conscientiousness (dutifulness)	0.41	Medium	0.00
Conscientiousness (orderliness)	0.31	Small	0.00
Extraversion (friendliness)	0.24	Small	0.00
Extraversion (excitement_seeking)	-0.21	Small	0.00
Emotional range (self_consciousness)	-0.16	Trivial	0.00
Openness (imagination)	-0.15	Trivial	0.00
Extraversion (gregariousness)	0.15	Trivial	0.00
Agreeableness (morality)	0.12	Trivial	0.00
Extraversion (cheerfulness)	-0.08	Trivial	0.00
Openness (artistic_interests)	0.04	Trivial	0.00
Agreeableness (altruism)	-0.04	Trivial	0.01

Extended Data Fig. 4. | **Founder Facet Footprint.** Features that distinguish founders (n=4.4k) from employees (n=6k). Note all 30 Big Five facets are significant at $p < 0.05$; however, Artistic Interests and Altruism are not significant at $p < 0.01$.

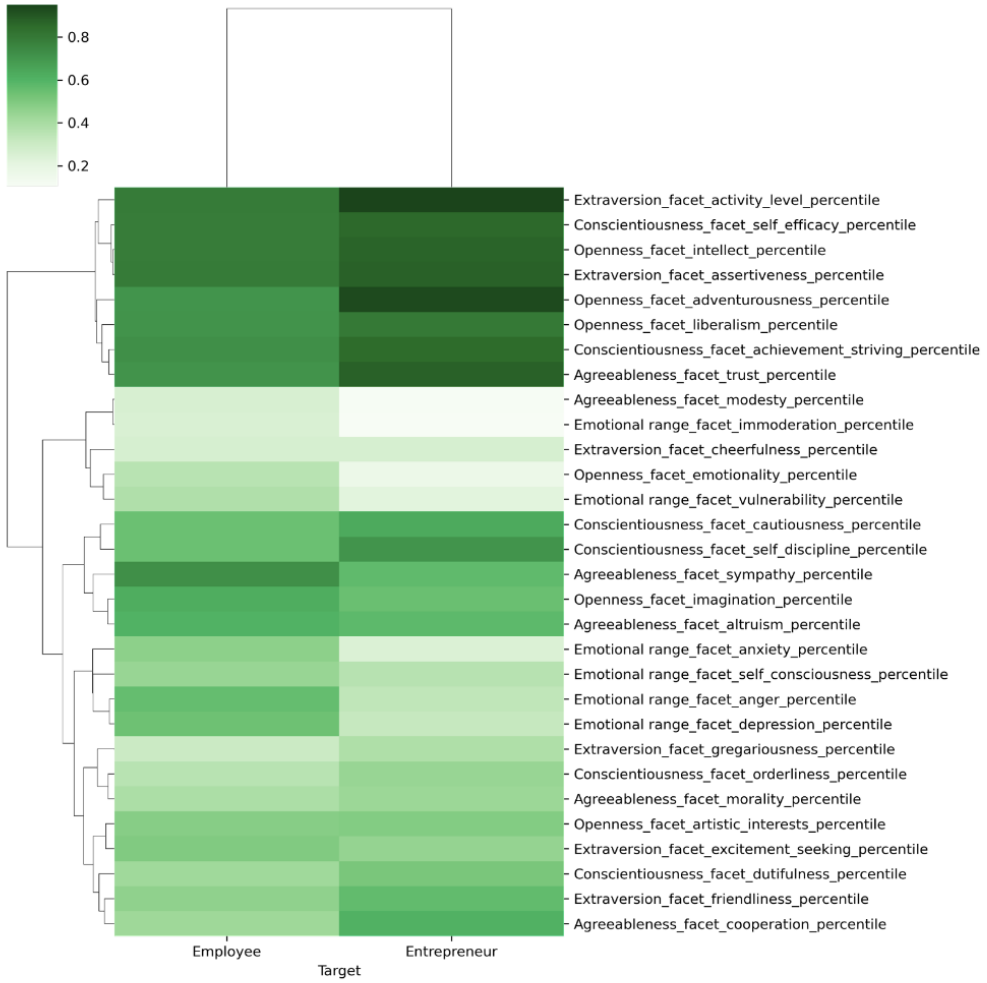

Extended Data Fig. 5. | **Entrepreneurial Personality Facets** The heatmap visualises the median values of 30 facets of two samples, which intuitively and comprehensively compare all personality traits and patterns. From the heatmap, it could be observed that the difference in median values of the two groups on adventurousness (Openness), activity level (Extraversion) and modesty (Agreeableness) are relatively large.

A.3 Clustering of founder personalities

In this part of the Supplementary Information, we provide additional statistics and analyses on the empirical clustering of founder personalities. Extended Data Figure 6 shows the clustering tendency of the founder personality data. Extended Data Figure 7 shows the dendrogram of the hierarchical clustering algorithm that was used to identify the groups in the data set. Extended Data Figure 8 presents statistics on the optimal number of clusters in the data and the frequency count of founders in each cluster. Extended Data Figure 9 lists the personality attributes of the occupation tribes corresponding most closely to the founder personality clusters. Extended Data Figure 11 investigates the robustness of the founder personality clusters against resampling.

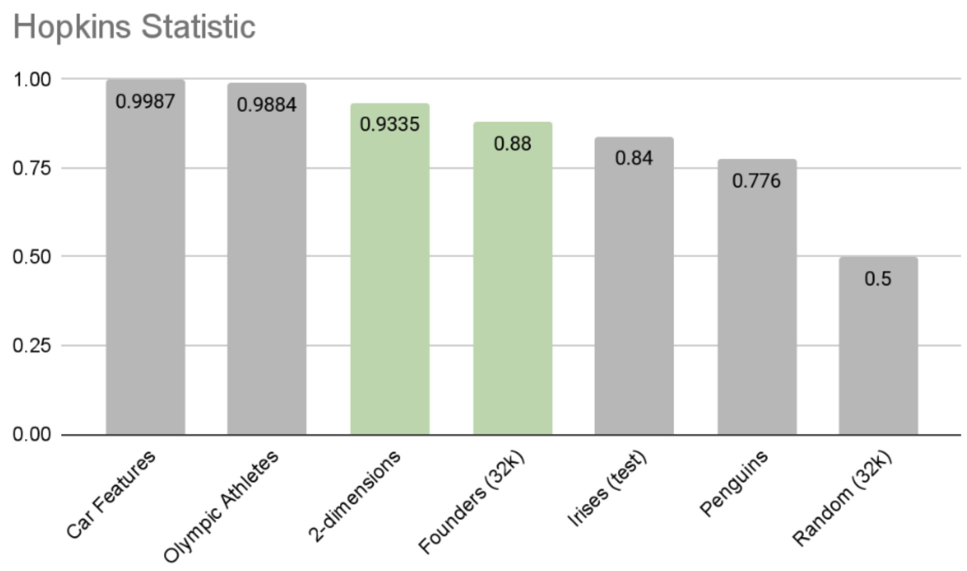

Extended Data Fig. 6. | **Clustering Tendency Startup Founders by Personality Feaures** Hopkins index (above) measures of the Founders inferred personality traits data, and 2-dimensions data (dimensionality reduction of Founders data) compares favourably to other famous test data sets (Irises; Penguins, Olympic Athletes) that are known to have explicit classes in the data — different breeds of Penguins, various species of Iris flowers and Olympic athletes who have qualified for different categories of events.

To better understand the unique personality characteristics of each of the six different clusters of founders and co-founders we:

1. **Analysed the personality footprints of each cluster.** We examined the distinctive personality traits of each group. We identified which clusters were home to the maximums in each of the 30 personality facets. We created a heat map revealing the complete personality footprint of each of the six types (Fig. 1D of the main text).

2. **Matched the occupation closest to the centre of each cluster** using the personality-occupation matrix from our previous research[?,?]. For each founder and co-founder, we found the closest corresponding *occupation tribe* for each based on personality similarity.

3. **Identified which of the eight occupation-tribes from previous research[?] each founder or co-founder belonged to.** Leveraging previous research, we then looked at the distribution of tribe membership of each founder within each cluster. Then we tallied the founders within each cluster by tribe to reveal the level of coherence or the extent to which most founders within each group belonged to one *occupation tribe*. This mapping to occupation tribes forms the basis for labelling the founder personality clusters #0 to #5 as Accomplishers, Engineers, Leaders, Developers, Operators, and Fighters.

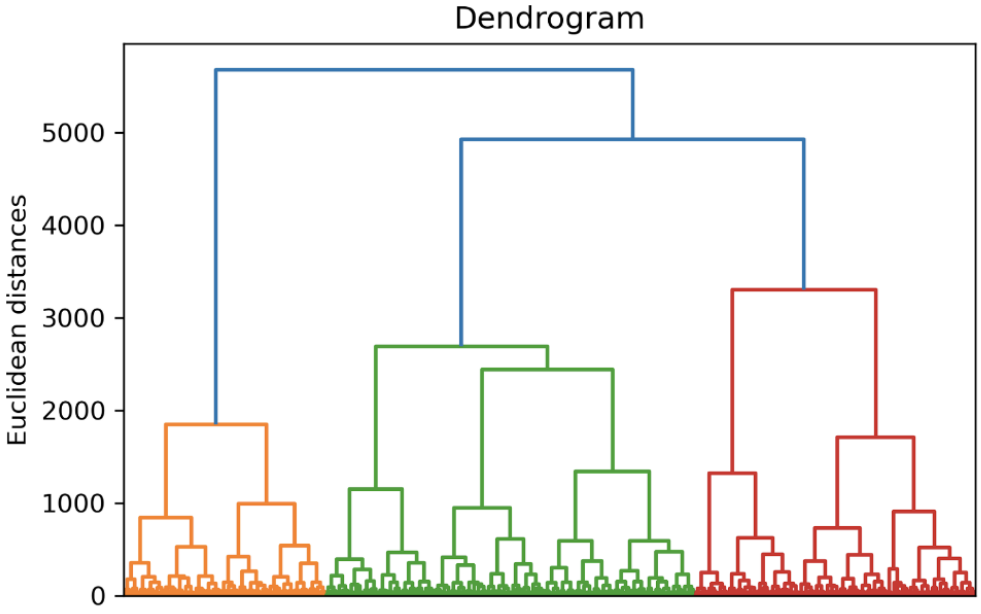

Extended Data Fig. 7. | **Hierarchical Clustering of Startup Founders by Personality Feaures** Hierarchical clustering was used to model potential clusters of startup founders by their personality features.

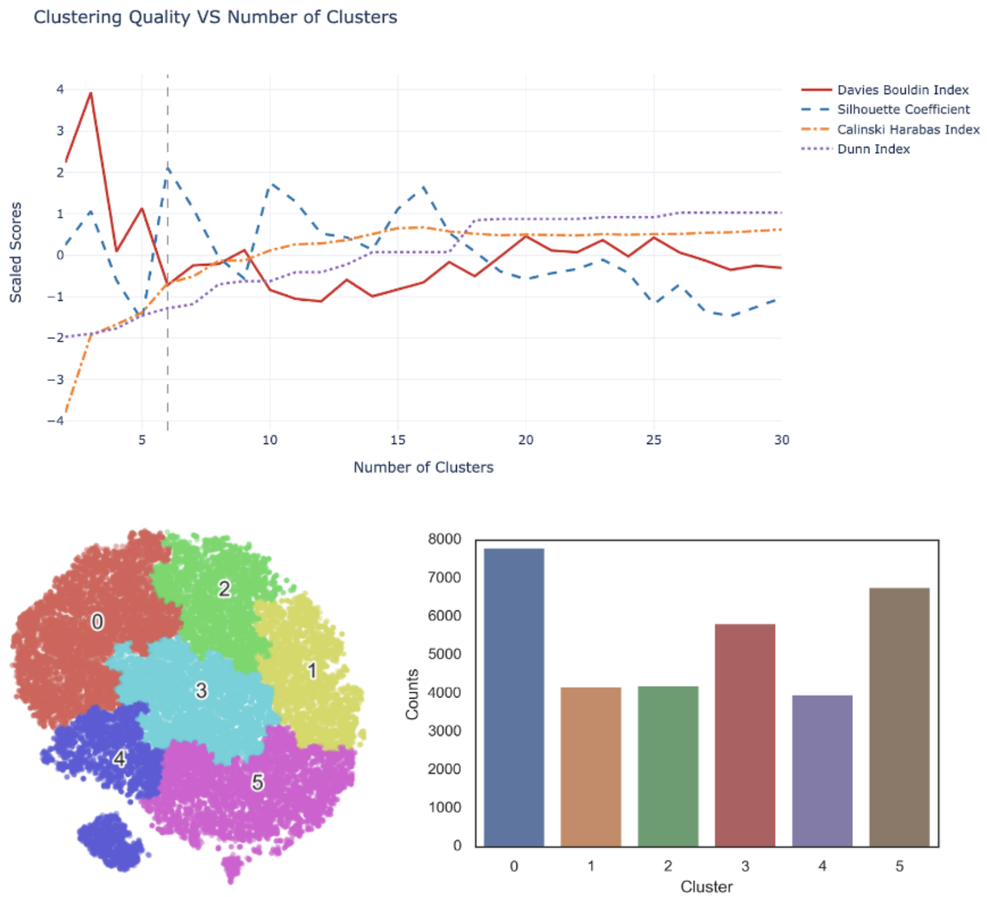

Extended Data Fig. 8. | **Optimum Number of Clusters of Types of Founders** Four different clustering quality indices were used to determine that there are optimally six clusters of startup founders. We labelled each of these #0, #1, #2, #3, #4 and #5 and produced the dendrogram, bar charts, and T-SNE plot above to demonstrate the hierarchy, adjacencies and distribution of all six clusters.

The Eight "Tribes"of Occupations	
**Tribe**	**Key personality attributes.**
**Leaders**	Adventurous, persistent, dispassionate, assertive, self-controlled, calm under pressure, philosophical, excitement-seeking & confident.
**Listeners**	Empathetic and compassionate - can understand others feelings, needs and suffering.
**Umpires**	Bold, altruistic, respectful of authority, outgoing & uncompromising. Pay attention to the fine details.
**Rebels**	Authority-challenging, imaginative and appreciative of art.
**Experts**	Curious, reserved, independent, trusting of others, self-assured, organised, carefree, deliberate, driven & energetic.
**Observers**	Compassionate, modest, consistent, cheerful, sociable, emotionally aware, laid-back, content
**Accomplishers**	Super organised & outgoing. Confident, Down-to-earth, Content, Accommodating, Mild-tempered & Self-assured.
**Fighters**	Spontaneous and impulsive. Tough, sceptical, and uncompromising.

Extended Data Fig. 9. | **Eight 'occupation tribes'** Tribes identified by McCarthy et al.[?] to empirically describe the fit between occupations and personality types.

Founder Type	Distinctive Personality Attributes	**Closest Occupation Fit** From occupation-personality maps
Fighters	Spontaneous and impulsive, tough, skeptical, and uncompromising.	Software Developer<br>Computer Engineer
Operators	Highest in conscientiousness in the facet of orderliness and high agreeableness in the facet of humility for founders in this cluster.	Bicycle Mechanic, Mechanic and Service Manager.
Accomplishers	Organised & outgoing. confident, down-to-earth, content, accommodating, mild-tempered & self-assured.	Chief Information Officer<br>Export Manager
Leaders	Adventurous, persistent, dispassionate, assertive, self-controlled, calm under pressure, philosophical, excitement-seeking & confident.	Executive Director<br>Medical Director
Engineers	Highest in openness in the facets of imagination and intellect.	Materials Engineer and Chemical Engineer.
Developers	“Middle child” cluster — no facets are maximums or minimums but it shares characteristics similar to fighters but higher in extraversion.	Application Developer and related technology roles such as Business Systems Analyst and Product Manager.

Extended Data Fig. 10. | **Details on the typology of founder personalities’** The Ensemble theory of Startup Success shows founders come in six types: Fighters, Operators, Accomplishers, Leaders, Engineers and Developers (FOALED), with each founder type having its own distinct personality facet footprint and closest occupation fit.

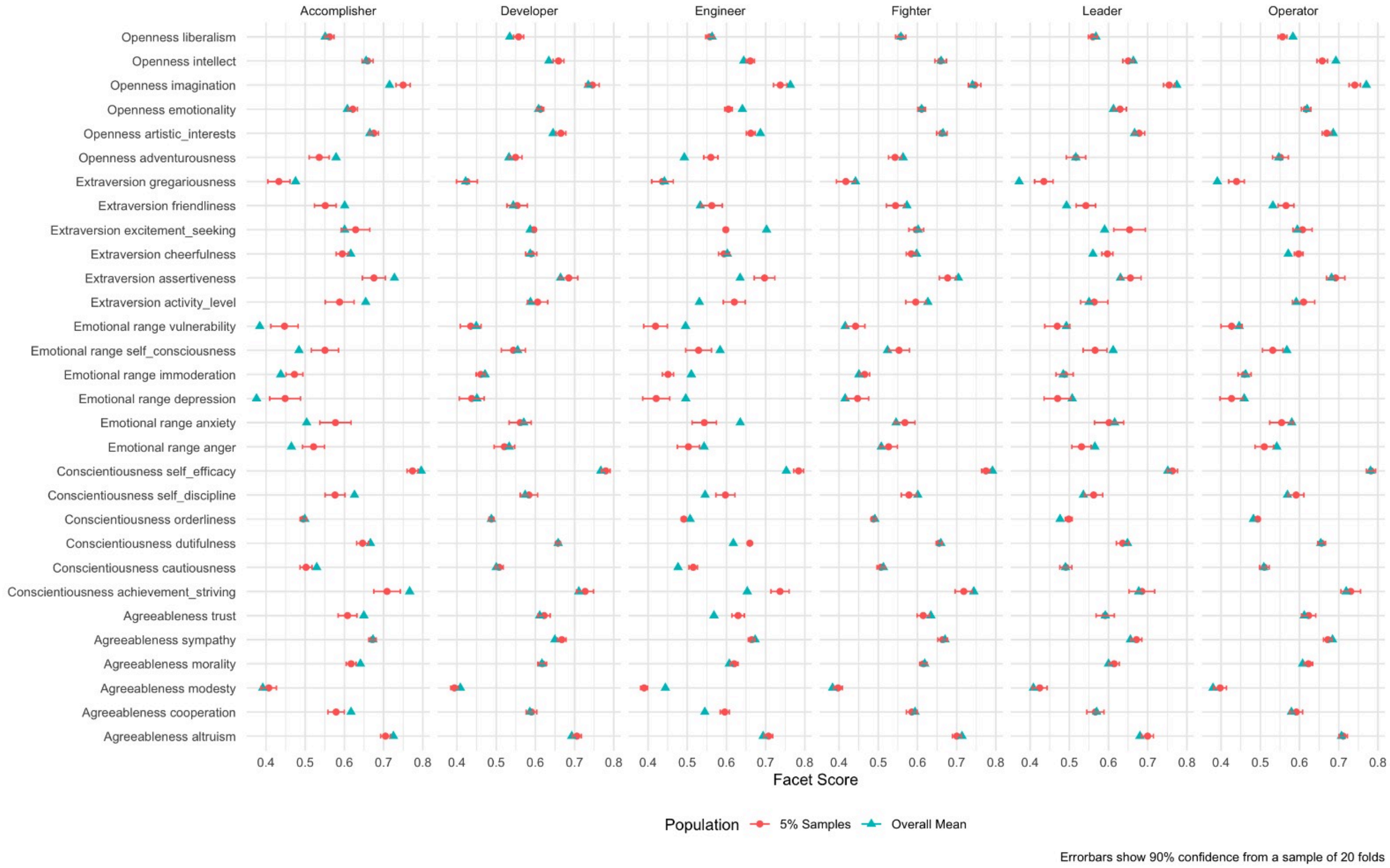

Extended Data Fig. 11. Robustness of personality facet clustering against resampling: the figure compares the overall mean value per facet in each cluster (green triangles) with the distribution of a 20-fold resampling of smaller samples from the founder data (red dot-whiskers) - in many cases the overall mean and the majority of the resampled means overlap; there are some facets with no overlap, but deviations tend to not be bigger than 0.1 points on the facet score scale.

A.4 Roles within startups

Information about personality traits not only helps to distinguish between individuals who tend to be founders of startup companies and employees, but it also correlates with the job role that founders will take in the startup companies they establish. For example, Extended Data Figure 12 shows the distribution of the six founder personality clusters by eight typical job roles in startup companies.

Two personality types are most clearly related to particular job roles. Accomplishers (#0) cluster in the roles of Chief Executive Officer, Chief Financial Officer, Chief Operating Officer, and Chief Marketing Officer. In contrast, Fighters (#5) are most prevalent in the roles of Chief Creative Officer, Chief Product Officer and Chief Technology Officer.

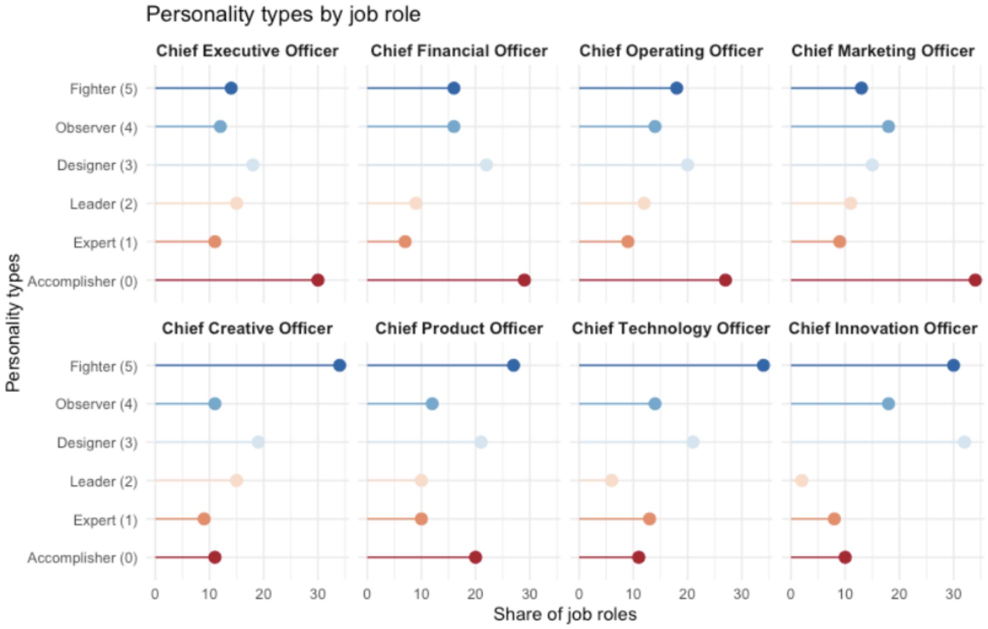

Extended Data Fig. 12. | **Occupations within Startups** While Accomplishers are often CEOs, CFOs or COOs, Fighters tend to be CTOs, CPOs, CCOs.

A.5 Data pre-processing for startup success prediction

To use the founder data from Crunchbase described above (32k profiles) for the success prediction, we needed to conduct several preparatory steps, which led to a reduction of the final number of observations as outlined in Extended Data Figure 13.

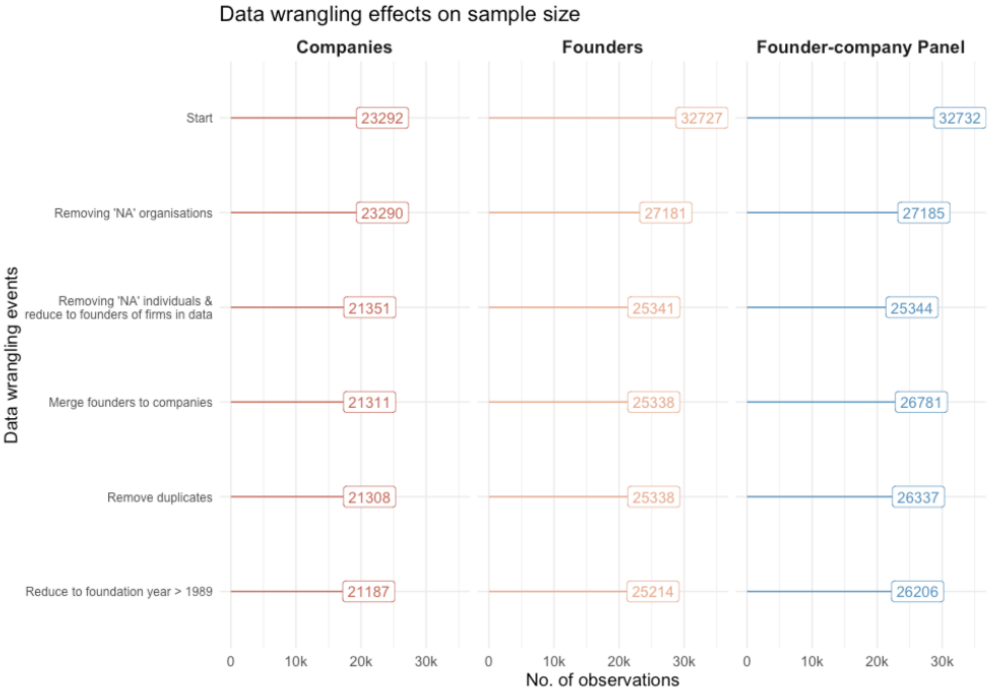

Extended Data Fig. 13. | **Data Wrangling for Multifactor Analysis** Effects of the data cleaning on the sample size. Five preparatory steps reduce the data set to 25,214 founders with inferred personality traits who have been involved in founding 21,187 startup companies.

The aim is to create a company-founder panel from the Crunchbase data based on exact founder names and company URLs as identifiers. Starting with 32,727 profiles corresponding to 23,292 companies, we removed organisations without names, reducing the data set to 27,181 founders and 23,290 companies. As a next step, we kept only those founders in the data set, founding the 23,290 companies in the data (via the 'founders' column), yielding 25,341 founders and 21,351 companies. Merging these founders with the companies led to a further reduction of the data set to 25,338 founders and 21,311 companies. The merging also resulted in some duplicates because of the identical names of some founders. These duplicates were removed by keeping only those company-founder combinations where the company of each potentially duplicated founder was mentioned either as their primary organisation or in their biography. This step did not affect the number of founders but reduced the number of companies by three, which could not unambiguously be assigned to any individual. As the last step, we removed companies that were founded before 1990, leading to a final data set of 25,214 founders involved in the foundation of 21,187 companies.

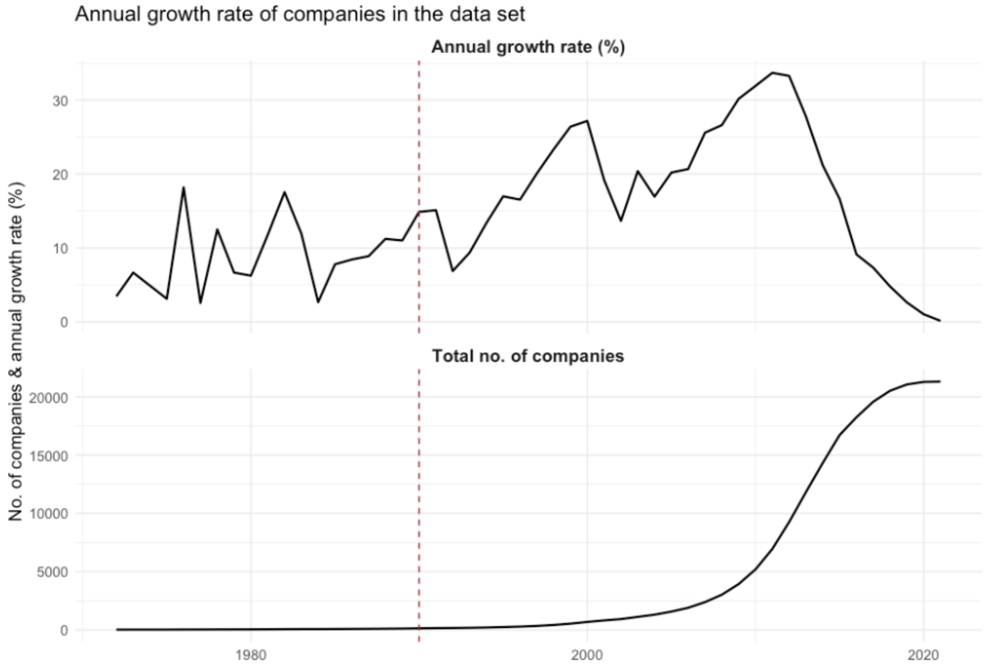

Extended Data Fig. 14. | **Foundation Year** Number of companies in the data set by foundation year. Following the approach taken by Bonaventura et al. (2020), we restrict the data set to those companies founded from 1990 onwards.

In reducing the data set to those companies that were founded from 1990 (see Extended Data Figure 14) onwards, we aimed at limiting the potential bias that could arise from having companies in the data set that cannot be considered as startup companies because of their age. Therefore, this additional restriction removes less than 0.6% of the companies in our data set.

In total, 3,442 of 21,187 companies (16%) in the data set have been successful according to the criterion used by Bonaventura et al.[?]. On average, successful companies needed 6.38 years to become successful (see Extended Data Figure 15).

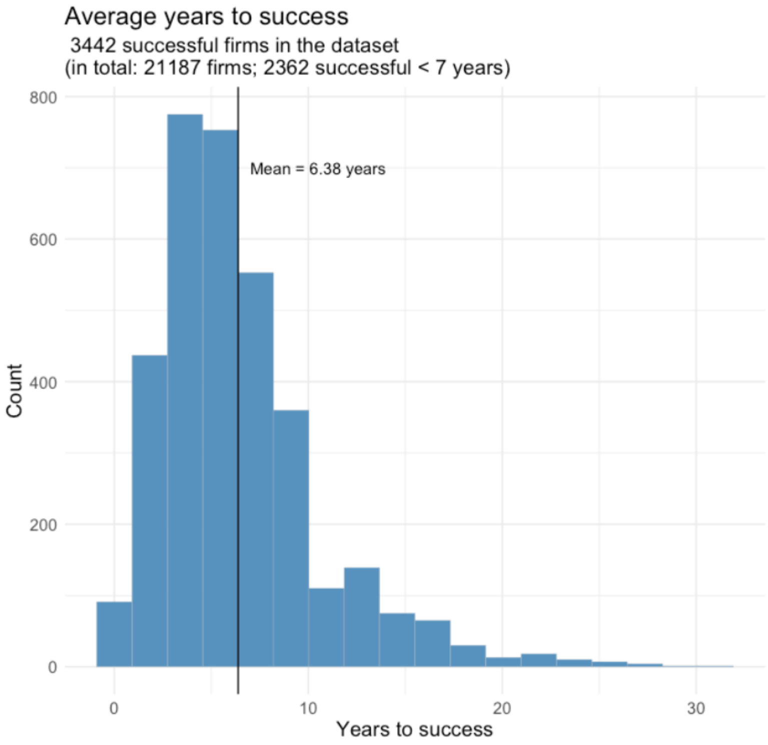

Extended Data Fig. 15. | **Years to success** Histogram of the variable 'average years to success' based on 3,442 successful firms in the data set. The mean time to success is 6.38 years.

The final data set is a panel with 26,202 observations, i.e. combinations of 25,214 founders involved in founding 21,187 companies. For each data point, we observe a total of 104 variables. Of those, 15 variables relate to the organisations and cover aspects such as a company identifier, description, industry categories, location, and foundation year. In addition, there are six variables related to success in the data: success date & type, success indicator variable, years to success since founded, and an indicator if success occurred within the first seven years after foundation. Eight variables refer to the founders: name, Twitter, biography, primary organisation, primary job role, gender, and social media; 75 variables present different characteristics of the inferred personalities: personality type, individual facets, Big Five traits, etc.

A.6 Startup success prediction

Besides the startup success prediction model presented in the main text, we provide additional analyses on the correlational patterns linking founder personalities to success here. Extended Data Figures 16 and 17 show that certain personality types tend to be associated more with successful startup companies than others.

In Extended Data Figure 18, we examine the gender differences between successful and unsuccessful founders. Extended Data Figure 19 compares the predictive performance of models that include a varying amount of information, and Extended Data Figure 20 shows the coefficient estimates of the final model displayed in the main text. Extended Data Figure 22 provides some evidence on mechanisms that might relate certain personality facets to startup success.

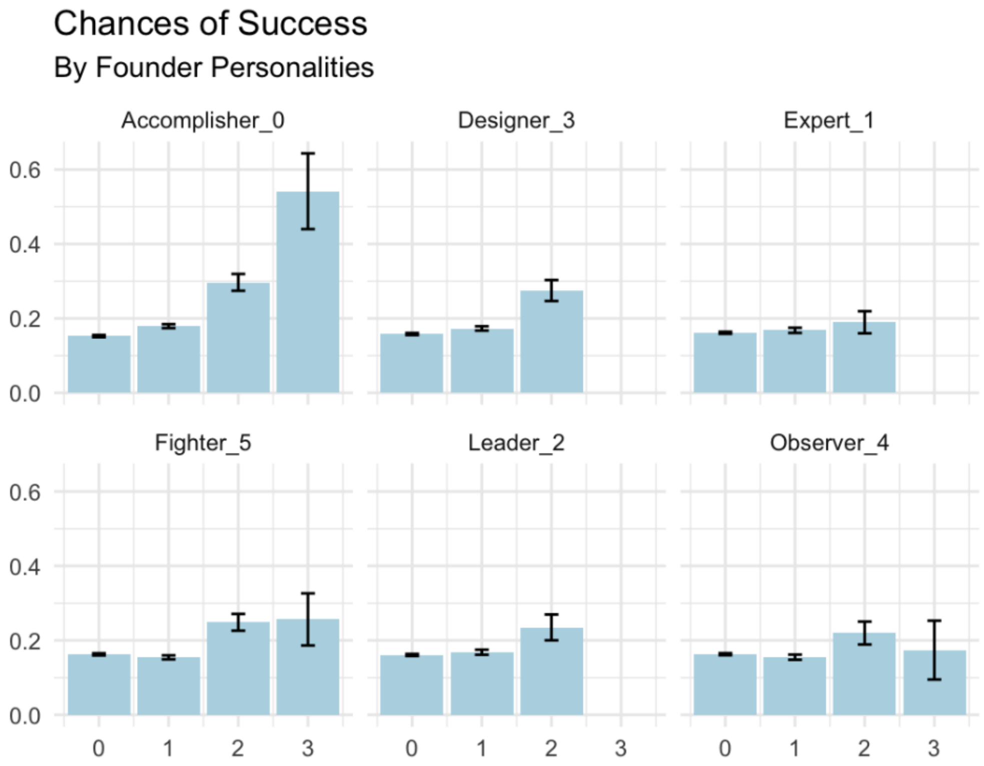

Extended Data Fig. 16. | **Founder Team and Success** Startups with multiple founders of the same personality types have higher chances of success.

Turning to gender differences in founder personalities, we compare the distribution of personality facets between successful and unsuccessful founders stratified by gender. While the multifactor modelling shows having male founders increases a startup's chance of success, this is likely primarily due to the significant gender bias in venture funding. In our data, female solo-founders received, on average, 20% less total funds than their male counterparts, while female co-founded teams received less than half the funding on average as all-male teams.

The amount of venture funds and angel investors specifically targeting female founders has proliferated since 2006, such as Women's Venture Capital Fund (Founded in Portland, Oregon in 2011); Female Founders Fund (founded in NY in 2014) and Halogen Ventures (founded in 2016 in LA). This will likely address some of these inequities over the current cohort of startups.

On the one hand, the correlations between external and internal factors and success, on the other hand, are visible when comparing different machine learning models that predict startup success. For example, Extended Data Figure 19 shows the predictive performance of six logistic regression models compared to a baseline random draw model. According to the recall Machine Learning Performance metric, the best-performing models (5) and (6) are more than 130% better than random draw. These models include several explanatory variables: foundation year, country, female indicator variable, the number of founders, as well as personality types (model 5) plus industries (model 6).

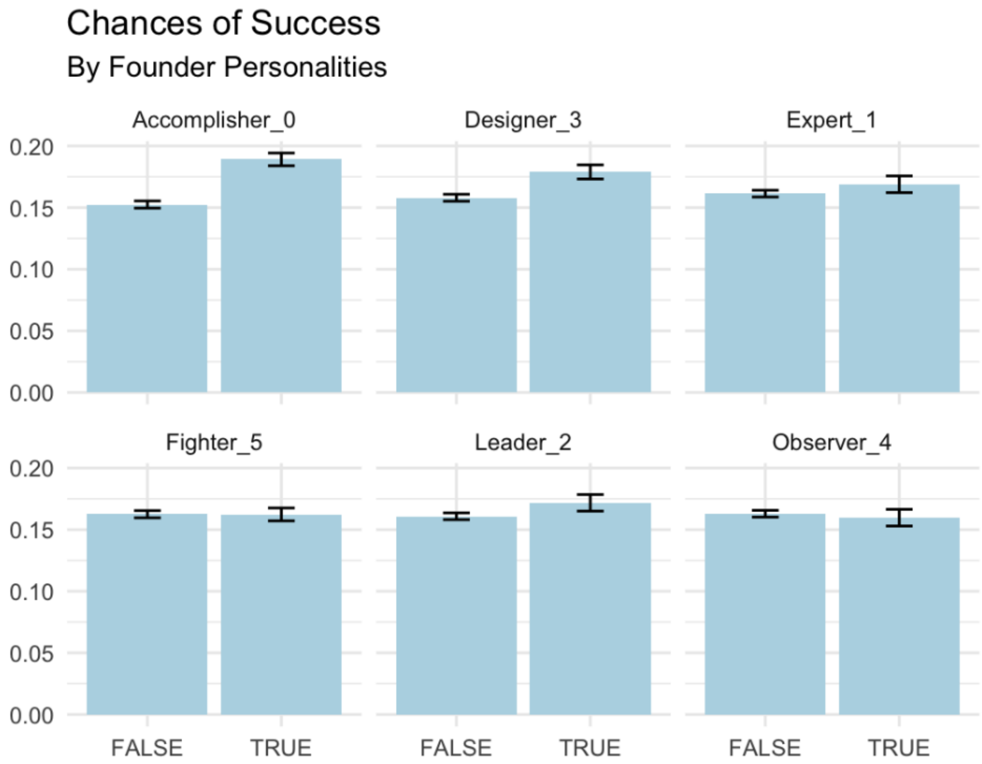

Extended Data Fig. 17. | **Founder types and success** Startups with specific founder personalities have higher chances of success - most significant for personalities of the 'Accomplisher' type.

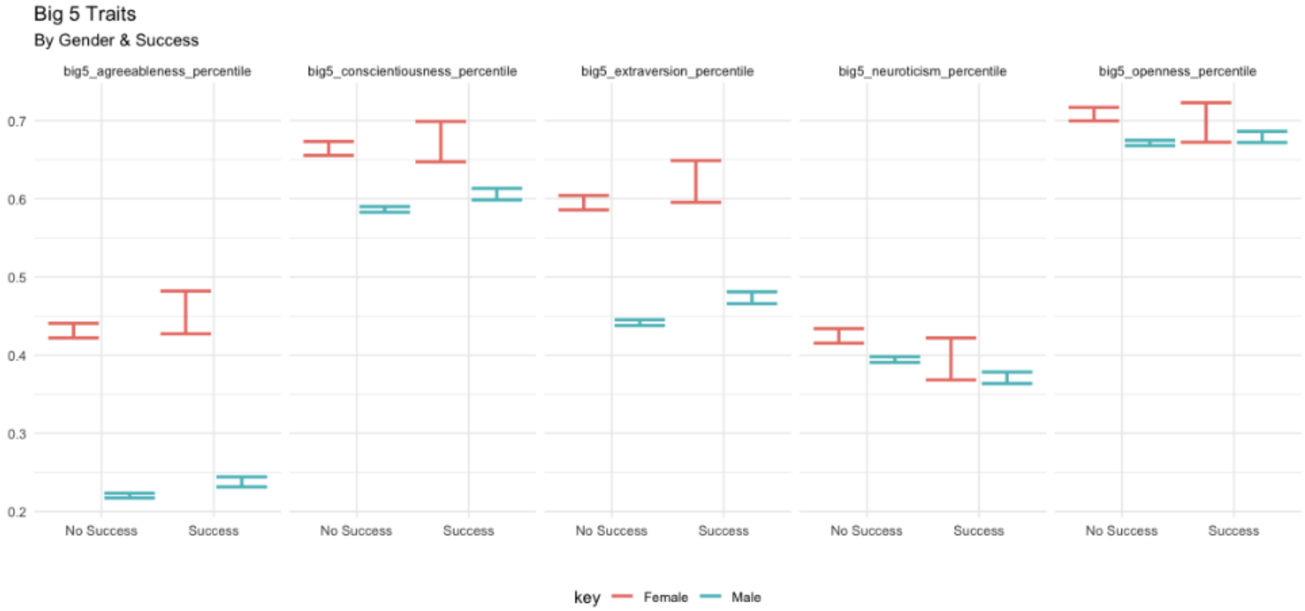

Extended Data Fig. 18. | **Gender bias in startups** We noted Successful female founders are more similar to the successful male founder.

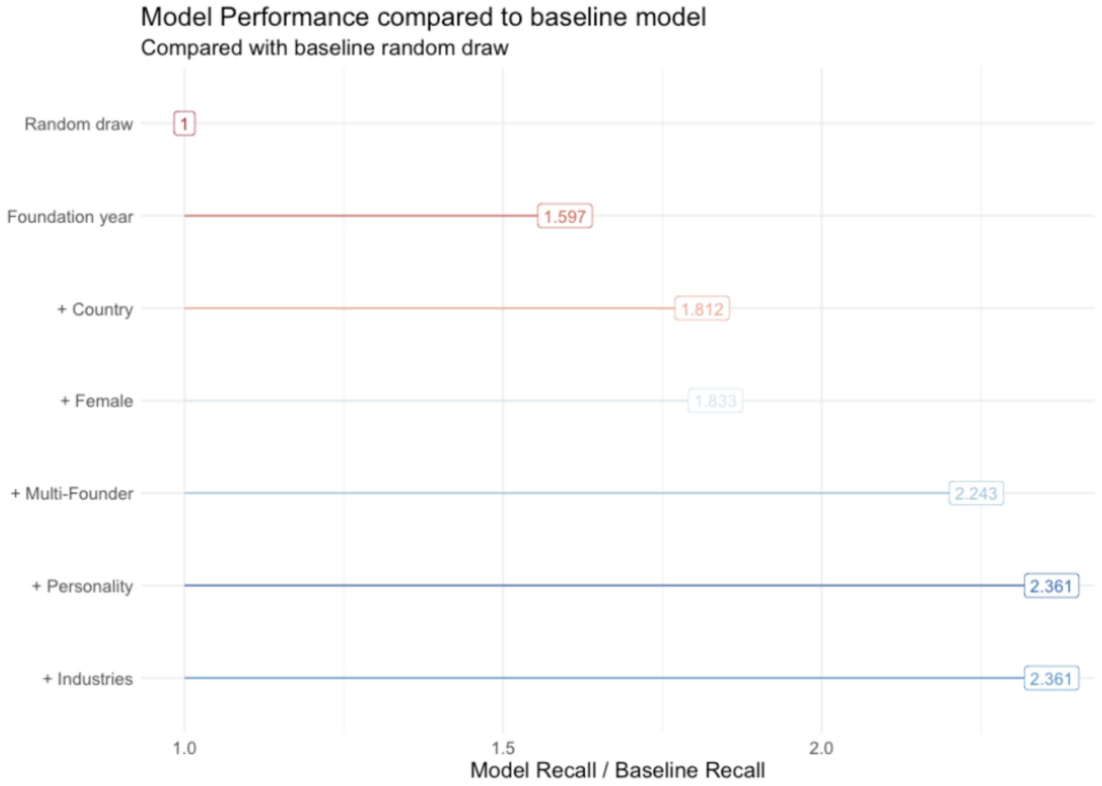

Extended Data Fig. 19. | **Recall Performance** Comparison of prediction performance according to Recall metric of different startup success prediction models (GLMs): (0) Null model of random draw, (1) simple model including only the foundation year as a predictive variable, (2) model 1 plus country, (3) model 2 plus female indicator variable, (4) model 3 plus count of a number of founders, (5) model 4 plus personality traits, (6) model 5 plus industries.

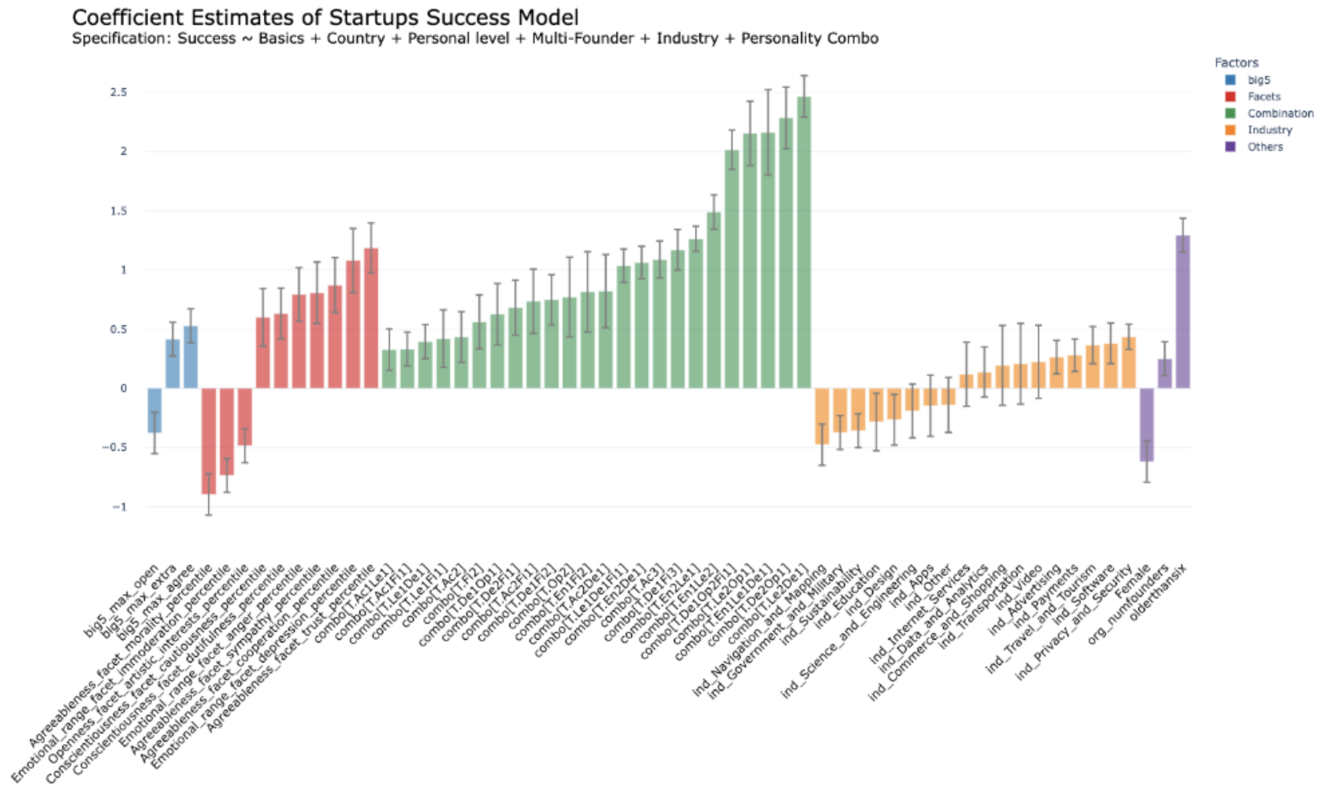

Extended Data Fig. 20. | **Ensemble Model** Multifactor modelling reveals the relative significance of different Firm-level, Founder-level, and Founder-Team level features on startup success and illustrates how personality-diverse larger teams have one of the most significant impacts on chances of success.

Features associated with Startup Success					
**Type of feature**	**Instance**	**coef**	**std err**	**p-value**	**odds ratio**
Team personality combinations	Leaders x 2, Developer x 1	2.56	1.17	0.03	12.90
Team personality combinations	Developers x 2, Operator x 1	2.14	0.77	0.01	8.46
Team personality combinations	Leaders x 1, Developer x 1, Engineer x 1	2.13	0.68	0.00	8.44
Startup Age	older than six years old	1.28	0.08	0.00	3.59
Team personality combinations	Accomplishers x 3	1.01	0.48	0.03	2.75
Personality of founders	Maximum Agreeableness (Big5)	0.47	0.12	0.00	1.60
Team personality combinations	Developer x 1, Operator x 1	0.46	0.22	0.03	1.59
Team personality combinations	Accomplishers x 2	0.46	0.14	0.00	1.59
Industry	Privacy and Security	0.46	0.09	0.00	1.58
Industry	Software	0.36	0.05	0.00	1.44
Industry	Payments	0.35	0.12	0.00	1.41
Team size	Number of founders	0.26	0.02	0.00	1.30
Industry	Transportation	0.22	0.09	0.02	1.25
Industry	Advertising	0.22	0.09	0.02	1.25
Industry	Data and Analytics	0.21	0.07	0.00	1.24
Industry	Commerce and Shopping	0.14	0.06	0.01	1.15
Industry	Information Technology	0.14	0.05	0.01	1.15
Industry	Internet Services	0.12	0.05	0.01	1.13
Industry	Other	-0.17	0.06	0.00	0.84
Industry	Apps	-0.18	0.07	0.02	0.84
Team personality combinations	Developer x 1	-0.18	0.08	0.02	0.84
Industry	Community and Lifestyle	-0.18	0.09	0.04	0.84
Team personality combinations	Fighter x 1	-0.24	0.09	0.01	0.79
Industry	Education	-0.24	0.09	0.00	0.78
Team personality combinations	Operator x 1	-0.27	0.09	0.00	0.76
Industry	Design	-0.31	0.10	0.00	0.73
Industry	Navigation and Mapping	-0.51	0.18	0.01	0.60

Note: only features with $p < 0.05$ tabled.

Extended Data Fig. 21. | **Coefficient estimates and odds ratios in the Ensemble Model** The table shows the coefficient estimates, standard errors, p-values, and odds ratios of several features in the Ensemble Model.

Big 5 (Facet) Higher/Lower	How people with this trait may be more successful as founders	Examples of successful startup founders who exhibit this trait
Openness (adventurousness) Higher	Founders with this trait may be more willing to take risks and explore new opportunities, which could lead to innovation and growth for the company.	Elon Musk, the founder of SpaceX and Tesla, is known for his willingness to take risks and explore new opportunities in industries such as space exploration and electric cars.
Agreeableness (modesty) Lower	Founders with this trait may be more confident and assertive in promoting their ideas and vision for the company, which could help them attract investors, customers, and employees.	Steve Jobs, the co-founder of Apple, was known for his confidence and assertiveness in promoting his vision for the company.
Extraversion (activity_level) Higher	Founders with this trait may have high levels of energy and drive, which could help them to accomplish more in less time. They may also be more likely to take on multiple projects and responsibilities, which could lead to growth and innovation for the company.	Richard Branson, the founder of Virgin Group, is known for his high energy and drive, which has helped him to build a successful business empire.
Emotional range (anxiety) Lower	Founders with this trait may be more resilient in the face of challenges and setbacks, which are common in the early stages of building a company. They may also be better able to manage stress and make clear-headed decisions under pressure.	Jeff Bezos, the founder of Amazon, is known for his ability to remain calm under pressure and make clear-headed decisions even in challenging situations.
Emotional range (immoderation) Lower	Founders with this trait may be more disciplined and able to resist temptation, which could help them to make better decisions for the long-term success of the company. They may also be better able to manage their emotions and avoid impulsive actions that could harm the business.	Mark Zuckerberg, the co-founder of Facebook, is known for his discipline and focus, which has helped him to build one of the most successful tech companies in the world.
Agreeableness (trust) Higher	Founders with this trait may be more likely to build strong relationships with employees, customers, and investors based on mutual trust and respect. This could help to create a positive work environment and foster loyalty and commitment.	Larry Page and Sergey Brin, the co-founders of Google, are known for their ability to build strong relationships with employees and foster a positive work environment based on mutual trust and respect.

Extended Data Fig. 22. Potential mechanisms on how personality might affect startup success.
